# How pattern formation in ring networks of excitatory and inhibitory spiking neurons depends on the input current regime

**DOI:** 10.3389/fncom.2013.00187

**Published:** 2014-01-07

**Authors:** Birgit Kriener, Moritz Helias, Stefan Rotter, Markus Diesmann, Gaute T. Einevoll

**Affiliations:** ^1^Department of Mathematical Sciences and Technology, Norwegian University of Life SciencesÅs, Norway; ^2^Institute of Neuroscience and Medicine (INM-6) and Institute for Advanced Simulation (IAS-6), Jülich Research Centre and JARAJülich, Germany; ^3^Faculty of Biology, University of FreiburgFreiburg, Germany; ^4^Bernstein Center Freiburg, University of FreiburgFreiburg, Germany; ^5^Medical Faculty, RWTH Aachen UniversityAachen, Germany

**Keywords:** pattern formation, spiking neurons, linear model, mean-driven, fluctuation driven, ring networks, small-world networks

## Abstract

Pattern formation, i.e., the generation of an inhomogeneous spatial activity distribution in a dynamical system with translation invariant structure, is a well-studied phenomenon in neuronal network dynamics, specifically in neural field models. These are population models to describe the spatio-temporal dynamics of large groups of neurons in terms of macroscopic variables such as population firing rates. Though neural field models are often deduced from and equipped with biophysically meaningful properties, a direct mapping to simulations of individual spiking neuron populations is rarely considered. Neurons have a distinct identity defined by their action on their postsynaptic targets. In its simplest form they act either excitatorily or inhibitorily. When the distribution of neuron identities is assumed to be periodic, pattern formation can be observed, given the coupling strength is supracritical, i.e., larger than a critical weight. We find that this critical weight is strongly dependent on the characteristics of the neuronal input, i.e., depends on whether neurons are mean- or fluctuation driven, and different limits in linearizing the full non-linear system apply in order to assess stability. In particular, if neurons are mean-driven, the linearization has a very simple form and becomes independent of both the fixed point firing rate and the variance of the input current, while in the very strongly fluctuation-driven regime the fixed point rate, as well as the input mean and variance are important parameters in the determination of the critical weight. We demonstrate that interestingly even in “intermediate” regimes, when the system is technically fluctuation-driven, the simple linearization neglecting the variance of the input can yield the better prediction of the critical coupling strength. We moreover analyze the effects of structural randomness by rewiring individual synapses or redistributing weights, as well as coarse-graining on the formation of inhomogeneous activity patterns.

## 1. Introduction

Understanding the dynamics of neuronal networks and its dependence on connection topology, weight distribution or input statistics is essential to understand network function. One very successful field in uncovering structure-dynamics relationships are so-called neural field models (see e.g., Beurle, 1956; Wilson and Cowan, 1972; Amari, 1977; Ermentrout and Cowan, 1979a; Ben-Yishai et al., 1995; Ermentrout, 1998; Coombes, 2005). In these models the positions of neurons in space are substituted by densities and their respective coupling is described by coupling kernels depending on pairwise spatial distance. Given certain symmetries such as translation invariance, and homogeneous input the resulting non-linear dynamics is often reduced to a low-dimensional system that can be studied analytically. Such systems can produce various spatio-temporal phenomena, such as traveling waves, activity bumps and formation of periodic patterns, which can be linked to activity in biological systems, e.g., visual hallucinations, feature selectivity, short term memory, or EEG rhythms (see e.g., Ermentrout, 1998; Coombes, 2005 and references therein).

However, when thinking of actual neuronal tissue, the symmetry requirements and continuity assumptions apply on a rather macroscopic scale, when neuron densities are high and heterogeneities are negligible. Moreover, such rate-based models cannot fully resolve the statistics of synaptic input currents, but it is well-known that neurons and neuronal populations react quite strongly to changes in the statistics of incoming currents in terms of mean, auto- and cross-covariance (see e.g., Mainen and Sejnowsky, 1996; Silberberg et al., 2004; Fourcaud-Trocmé and Brunel, 2005; De la Rocha et al., 2007; Boucsein et al., 2009; Tchumatchenko and Wolf, 2011). Finally, it is often unclear how to quantitatively interpret the parameters of abstract field models, especially when compared to spiking network simulations.

Along these lines, Usher et al. (1995) demonstrated numerically how periodic pattern-formation takes place in a two-dimensional toroidal network of excitatory and inhibitory pulse-coupled integrate-and-fire oscillators. The bifurcation occurs when the relative surround inhibition reaches a certain threshold that is determined from the Fourier-transform of the effective coupling matrix (dispersion relation). In their study, Usher et al. (1995) moreover observed that the dynamics of the emerging pattern depends on the average external input current, with slowly diffusing hexagonally arranged activity bumps for low input current, over stationary bumps for intermediate input strength, to coherently moving bumps for strong external input.

In a later study, Bressloff and Coombes (1998, 2000) showed rigorously how periodic orbits in networks of pulse-coupled inhibitory and excitatory neurons with Mexican-hat topology indeed undergo a discrete Turing-Hopf-bifurcation, if the system is coupled strongly enough.

Roxin et al. (2005) studied a neuronal field model with particular emphasis on the role of delays. Similar to earlier studies of neuronal field models, (see e.g., Ben-Yishai et al., 1995; Compte et al., 2000; Shriki et al., 2003) neurons were distributed along a ring and coupling was distance-dependent. In particular, the coupling kernel was of the form *J*(|*x* − *y*|) = *J*_0_ + *J*_1_ cos(*x* − *y*), where *x* and *y* are neuron positions in space and *J*_0_ and *J*_1_ are coupling strength coefficients. Depending on the choice of *J*_0_, *J*_1_ and the delay the system can show a plethora of activity states such as traveling waves, stationary or oscillatory bumps, and also aperiodic behavior. However, this choice of interaction between two neurons is rather qualitative. It is usually symmetric and changes sign with distance, properties that are not in line with the biology of individual neurons and synapses.

Thus, to compare the field-model results to simulations of networks of excitatory and inhibitory spiking neurons, Roxin et al. (2005) set up a model where the same number of excitatory and inhibitory neurons were uniformly distributed across two rings and were coupled by sinusoidally modulated connection probability. This set-up resulted in an effectively one-dimensional model where at each position excitation and inhibition combine to a net-coupling comparable to the field model, and spatio-temporal patterns qualitatively matched the field-model predictions. The parameters in the spiking model were chosen with regard to biophysically plausible values, and the non-linearity of the current-to-rate transduction in the field model was chosen as threshold linear in an *ad-hoc* manner, however a direct quantitative mapping between neuronal network and field model was not tried.

Here, we investigate the dynamics of identical leaky integrate-and-fire neurons that are arranged on ring and grid networks where both inhibitory and excitatory neurons are uniquely assigned positions in space rather than representing a density. No two neurons can be at the same position, and this is thus a step toward spatially embedded networks of spiking neurons instead of neuronal populations with possibly ambiguous coupling weights. Instead, individual neurons have a distinctive identity in that they act either excitatorily or inhibitorily on all their post-synaptic targets (Dale's principle) and the coupling between any two neurons is usually not symmetrical. This is important since neglecting the identity of a neuron can lead to dramatically different dynamics (Kriener et al., 2008, 2009). If the system is translation-invariant, a Turing-instability occurs for a critical synaptic coupling strength *J*_*c*_, such that there is a bistability between the spatially homogeneous firing rate distribution and an inhomogeneous periodic spatial pattern, similar to what was described before (Usher et al., 1995; Bressloff and Coombes, 1998, 2000).

We find that the value of *J*_*c*_ depends strongly on the statistics of the neuronal input: if neurons are mean-driven, i.e., receive suprathreshold input, the instability occurs for much smaller coupling strength than when neurons are in the strongly fluctuation-driven regime, where the mean input is subthreshold and spikes are evoked by spontaneous suprathreshold fluctuations. The latter is the key mechanism in explaining asynchronous-irregular firing in balanced neuronal networks.

In assessing stability of an invariant network state to perturbations the usual *modus operandi* is to linearize around the fixed point (see e.g., Ermentrout and Cowan, 1979a,b, 1980; Usher et al., 1995; Bressloff and Coombes, 1998, 2000; Coombes, 2005), implying crucially the derivative of the neuronal input-output function. In the fluctuation-driven regime the input-output function depends on both the mean and variance of the input current and thus both respective derivatives are expected to influence the quantitative prediction of linear fixed point stability (Amit and Brunel, 1997; Tetzlaff et al., 2012; Helias et al., 2013). Indeed, in the strongly fluctuation-driven regime, such that the standard-deviation of the input current is much larger than the average distance to firing threshold, the correct critical coupling strength is derived by linearization with respect to both mean and variance around the fixed point. In the mean-driven regime, on the other hand, the linearization becomes independent on the input current variance, and the derivative with respect to the mean is constant over wide ranges of working points implying independence on the exact location of the rate fixed point. In this regime pattern formation typically occurs for considerably smaller coupling strength than in the fluctuation-driven regime. Interestingly, if the input current is in an intermediate regime with both moderate subthreshold input mean, yet considerable variance the linearization independent of the input variance can give a better estimate than the one taking it into account. We will see that usually, the true critical coupling strength will lie inbetween both estimates, with *J*_*c*_ generally closer to the mean-driven regime prediction.

Moreover, in order to relate our ring model to the earlier findings of pattern formation in the classical Ermentrout-Cowan networks (Ermentrout and Cowan, 1979a,b, 1980) we also introduce a coarse-grained network that maps to a two-dimensional system which is formally identical to Ermentrout-Cowan networks, but lacks some of the details of the full, effectively five-dimensional ring network considered here.

Finally, we study the role of robustness of pattern formation to randomness: first, structural randomness is caused by the introduction of short-cuts which move the ring topology into the so-called small-world regime (Watts and Strogatz, 1998); secondly, we also discuss the effect of randomness in the weight distribution introduced by lifting the constraint that neurons are either fully excitatory in their action or fully inhibitory (Dale's principle).

## 2. Materials and methods

### 2.1. Neuron and network model

We study ring networks of *N* leaky integrate-and-fire (LIF) neurons with current-based synapses. *N*_*E*_ = β*N*, β ∈ [0, 1], of all neurons are excitatory, the residual *N*_*I*_ = *N* − *N*_*E*_ neurons are inhibitory. The membrane potential *V*_*i*_(*t*) of neuron *i* is governed by the differential equation

(1) $$\tau_{\text{m}}\frac{dV_{i}(t)}{dt}=-V_{i}(t)+RI_{\text{s,i}}(t-d)+RI_{\text{x,i}}(t),$$

where the membrane potential *V*_*i*_(*t*) is reset to *V*_res_ whenever it reaches the threshold potential *V*_thr_ and a spike is emitted. The neuron is then refractory for a time τ_ref_. τ_m_ denotes the membrane time constant, $RI_{\text{s,i}}(t-d)=\tau_{\text{m}}\sum_{j\;=\;1,\;i\;\neq \;j}^{N}W_{ij}s_{j}(t-d)$, where *I*_s_ is the input current received from the local network, and *s*_*j*_(*t*) = ∑_*k*_ δ(*t* − *t*_*j*, *k*_) is the spike train produced by some neuron *j*. *d* is the transmission delay, and *W* is the coupling matrix, with entries that are either zero (no synapse present), *J*_E_ = *J* (excitatory synapse) or *J*_I_ = −*gJ* (inhibitory synapse). To keep spiking activity from entering a high rate state, we assume that the recurrent input *I*_s_ is net inhibitory by choosing *g* > *N*_*E*_/*N*_*I*_.

*I*_x_ denotes the external input current that is modeled as stationary Poisson noise injected via current-based synapses of strength *J*_x_ (for details, see below), and *R* is the membrane resistance. Such leaky integrate-and-fire neurons were extensively studied in various input (see e.g., Amit and Tsodyks, 1991; Amit and Brunel, 1997; Lindner, 2004; Fourcaud-Trocmé and Brunel, 2005; De la Rocha et al., 2007; Vilela and Lindner, 2009; Helias et al., 2010) and network settings, especially in the context of balanced random networks (Brunel, 2000; Tetzlaff et al., 2012). Here, we consider ring networks of LIF neurons, such that each fifth neuron is inhibitory and the coupling matrix *W* is given by (cf. Figure 3A)

(2) $$W_{ij}=\{\begin{array}{ll} J & \text{if }j\text{ excitatory, }|i-j|\mathrm{mod}N\leq \kappa /2\text{ and }i\neq j\\ -gJ & \text{if }j\text{ inhibitory},\;|i-j|\mathrm{mod}N\leq \kappa \text{/2 and }i\neq j\text{.}\\ 0 & \text{otherwise} \end{array}$$

Thus, each neuron is connected to its κ nearest neighbors, where we assume κ = 0.1*N*. We note that the network layout chosen here is motivated by an actual embedding of individual neurons into some space, in contrast to earlier studies of pattern formation in spiking neuron networks where inhibitory and excitatory neurons are often distributed in equal numbers along two identical rings, and where the main difference lies in the spatial interaction ranges. These systems are usually motivated by population-density descriptions and can often be reduced to a two-dimensional model (see e.g., Ben-Yishai et al., 1995; Compte et al., 2000; Shriki et al., 2003; Roxin et al., 2005). As we will demonstrate in section 3.2, the network studied here leads to an effectively five-dimensional model. In section 3.4 we will discuss a reduction to a two-dimensional model that however already deviates noticeably in its details from the full five-dimensional network.

### 2.2. Constant external input in the mean-driven and intermediate regime

In the case of the mean-driven (μ[*RI*] ≥ *V*_thr_ − *V*_res_) and intermediate regimes, which will be discussed later, the external current is purely excitatory, such that *RI*_x_(*t*) = τ_m_*J*_x_*s*_x_(*t*) with *J*_x_ > 0 and rate ν_X_ = E[*s*_x_(*t*)], where E[.] denotes the expectation value. To quantify the effective strength of the external input, we express ν_X_ = ηθ/*J*_x_τ_m_, i.e., the parameter η gives the strength of the external drive in terms of the excitatory rate necessary to reach threshold. Assuming stationary spiking activity with rate ν_*o*_ for both the excitatory and inhibitory population, i.e., ν_E_ = E[*s*_E_(*t*)] = ν_*o*_ and ν_I_ = E[*s*_I_(*t*)] = ν_*o*_ respectively, the mean and variance of the total input *RI* = *R*(*I*_s_ + *I*_x_) are thus given by

(3) $$\begin{array}{l} \mu \left[R\left(I_{\text{s}}+I_{\text{x}}\right)\right]=\sum_{j\in \{\text{E,I,X}\}}\tau_{\text{m}}\nu_{j}J_{j}=\kappa \tau_{\text{m}}\nu_{o}J(\beta -g(1-\beta ))+\tau_{\text{m}}\nu_{\text{X}}J_{\text{x}}\\ \sigma^{2}\left[R\left(I_{\text{s}}+I_{\text{x}}\right)\right]=\sum_{j\in \{\text{E,I,X}\}}\tau_{\text{m}}\nu_{j}J_{j}^{2}=\kappa \tau_{\text{m}}\nu_{o}J^{2}(\beta +g^{2}(1-\beta ))+\tau_{\text{m}}\nu_{\text{X}}J_{\text{x}}^{2}. \end{array}$$

For stronger local coupling strength *J* the negative mean recurrent contribution μ[*RI*_s_] to the total input is stronger, while the variance σ^2^[*RI*_s_] increases. For the networks we consider here, for small *J* the network activity is thus typically driven by the mean μ[*RI*] of the total input, while for larger *J* the variance σ^2^[*RI*] has a notable impact. Moreover, the network firing rate ν_*o*_ depends on *J*.

### 2.3. External input adjusted to ensure constant total current in the strongly fluctuation-driven regime

The strongly fluctuation-driven regime is characterized by subthreshold mean total input, μ[*RI*] < *V*_thr_ − *V*_res_, but high variance σ^2^[*RI*], such that spikes are initiated by transient input fluctuations. To guarantee that we are in the high variance regime for all *J*, we inject excitatory and inhibitory external currents with rates ν_Ex_, ν_Ix_, such that the total input current mean and variance, i.e., μ[*RI*] and σ^2^[*RI*], stay the same when varying the local coupling strength *J*. This is achieved by adapting μ[*RI*_x_] = μ[*RI*] − μ[*RI*_s_] and σ^2^[*RI*_x_] = σ^2^[*RI*] − σ^2^[*RI*_s_] as a function of ν_Ex_, ν_Ix_. The external current injected into each local neuron by current-based synapses of strengths *J*_Ex_ = *J*_x_ and *J*_Ix_ = −*gJ*_x_ is thus given by

(4a) $$RI_{\text{x}}(t)=\tau_{\text{m}}J_{\text{x}}(s_{\text{Ex}}(t)-gs_{\text{Ix}}(t))$$

(4b) $$\text{with }\nu_{\text{Ex}}:=\text{E}[s_{\text{Ex}}(t)]=\frac{1}{\tau_{\text{m}}J_{\text{x}}(1+g)}\left(\frac{\sigma^{2}[RI_{\text{x}}]}{J_{\text{x}}}+g\mu [RI_{\text{x}}]\right)$$

(4c) $$\text{and }\nu_{\text{Ix}}:=\text{E}[s_{\text{Ix}}(t)]=\frac{1}{\tau_{\text{m}}J_{\text{x}}g(1+g)}\left(\frac{\sigma^{2}[RI_{\text{x}}]}{J_{\text{x}}}-\mu [RI_{\text{x}}]\right).$$

Because the variance of the recurrent input σ^2^[*RI*_s_] increases quickly with increasing *J*, the total variance σ^2^[*RI*]− in order to keep it fixed for all *J*− usually needs to be chosen quite large in order to avoid non-sensical rates ν_Ix_ < 0. This moreover implies that this approach of choosing the external input is not useful for the mean-driven scenario.

For all neurons we set θ = *V*_thr_ − *V*_res_ = 20 mV, τ_m_ = 20 ms, and *R* = 80 MΩ. The excitatory synaptic coupling strengths *J, J*_x_, the relative strength of inhibition *g*, as well as τ_ref_ and *d* will be specified individually. The model and parameters are listed in Table A1 in Appendix A1. All simulations were carried out with NEST (Gewaltig and Diesmann, 2007).

## 3. Results

**Figure 1:**
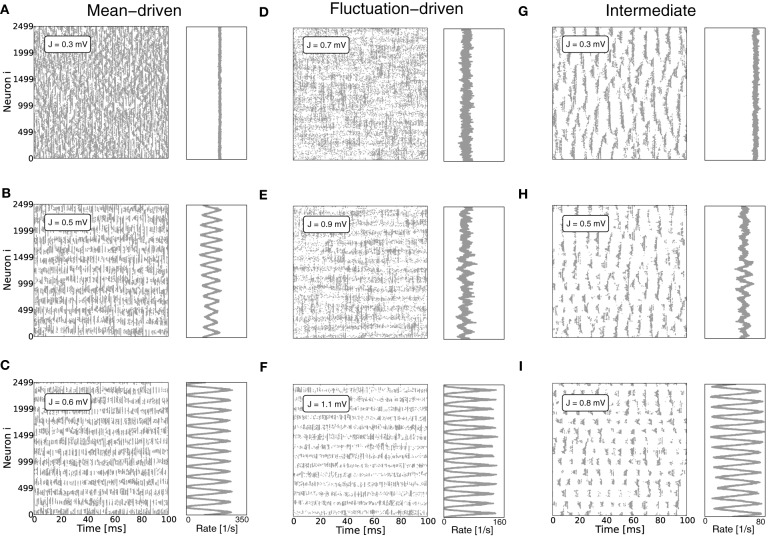
**Pattern formation in a ring network of 2500 neurons with *g* = 6 and varying absolute coupling strength parameter *J***. **(A–C)** Show the mean-driven case, where the external input is given by Poissonian input spikes with rate ν_x_ = 10θ/*J*_x_τ_m_ and amplitude *J*_x_ = 0.1 mV, resulting in an excitatory input current of amplitude *I*_x_ = 2500 pA. The critical coupling strength *J*_*c*_ for the spatially homogeneous rate distribution to become bistable with a spatially modulated pattern is around 0.5 mV. **(D–F)** Show the same network in the fluctuation driven regime, where mean and variance of the external input current *I*_x_ were chosen such that mean and standard deviation of the total input current *I* = *I*_x_ + *I*_s_ were fixed at μ[*RI*] = 5 mV and σ[*RI*] = 60 mV, cf. Equation (4). **(G–I)** Show the corresponding plots for an intermediate input regime, where the external input current was Poissonian with rate 3.5θ/*J*_x_τ_m_ and amplitude *J*_x_ = 0.1 mV, resulting in an input current of amplitude *I*_x_ = 875 pA. Here, refractory time constant τ_ref_ and delay *d* were chosen to be 0.1 ms to minimize loss of input current and avoid pronounced delay oscillations in the mean-driven case (Helias et al., 2013). As we will demonstrate in section 3.2, eigenvalue analysis predicts an instability of an eigenmode with non-zero wavenumber Λ_*c*_ > 0 (here, Λ_*c*_ = 13) for coupling *J*^md^_*c*_ ≈ 0.5 mV in the mean-driven case, and *J*^fd^_*c*_ ≈ 0.9 mV in the fluctuation-driven case. We see that already for *J* ≲ *J*_*c*_ there can be deviations from the spatially homogeneous mode Λ = 0 due to the fluctuating nature of the activity and selective amplification (Dayan and Abbott, 2001), especially in the fluctuation-driven case. For larger *J* the pattern becomes more prominent and finally some neurons fall completely silent **(C,F,I)**.

For small coupling strength *J* and large mean current input μ[*RI*] = E[*R*(*I*_x_ + *I*_s_)] the spiking dynamics of the ring networks is characterized by locally clustered activity on the spatial scale of the footprint κ, cf. Figures 1A,G (Kriener et al., 2009). For increasing *J*, however, a clear spatial pattern emerges, cf. Figures 1C,F. Since all neurons receive by construction statistically identical input, all neurons should fire at the same rate. But there is a distinct critical coupling strength *J*_*c*_ beyond which the homogeneous distribution of firing rates becomes unstable and the system enters a spatially inhomogeneous state after sufficient perturbation (Bressloff and Coombes, 1998). We note that the spatio-temporal spiking activity can be quite rich in networks with translation-invariant symmetry, as discussed for example in (Ben-Yishai et al., 1995; Usher et al., 1995; Bressloff and Coombes, 1998, 2000; Shriki et al., 2003; Roxin et al., 2005; Marti and Rinzel, 2013). Here, we do not systematically analyze the temporal aspects of the activity in terms of traveling waves or oscillations, but focus on the onset of periodic pattern formation in space as a function of coupling strength.

What determines this critical coupling strength *J*_*c*_ where the bistability of the homogeneous spatial rate distribution and the spatially modulated distribution occurs? We find that *J*_*c*_ depends strongly on the input regime the neurons operate in: if neurons are driven predominantly by the mean of the input current, i.e., if μ[*RI*] ≥ θ, we find that *J*_*c*_ is much smaller than if neurons are strongly fluctuation-driven, i.e., μ[*RI*] < θ with large enough variance of the input current to occasionally drive the membrane potential to threshold, cf. Figure 1.

In the following we present two different linear rate models of the integrate-and-fire dynamics that explain and predict the respective occurrence of the pattern formation.

### 3.1. Two linearizations for the self-consistent rate in networks of LIF-neurons

The analysis of the stability of spatially homogeneous activity dynamics to spatial perturbations follows a simple concept: first, the stationary, homogeneous state is determined in a mean-field approximation. In particular, in the diffusion limit, i.e., under the assumption that the coupling strength *J* is small compared to the distance between reset and threshold θ and that all neurons receive statistically identical input, the stationary firing rate ν_*o*_ of the neurons can be derived self-consistently as a function of mean and variance of the input current (a solution of the mean first-passage-time problem first derived by Siegert (Siegert, 1951), but also see e.g., Amit and Tsodyks, 1991; Brunel, 2000), such that

(5) $$\nu_{o}^{-1}=\tau_{\text{ref}}+\tau_{\text{m}}\sqrt{\pi }\int_{\frac{V_{\text{res}}-\mu_{o}}{\sigma_{o}}}^{\frac{V_{\text{thr}}-\mu_{o}}{\sigma_{o}}}\text{exp}\left[x^{2}\right](1+\text{erf}[x])dx,$$

where μ_*o*_ = ∑_*i* ∈ {E,I,X}_ *J*_*i*_ν_*i, o*_τ_m_ and σ^2^_*o*_ = ∑_*i* ∈ {E,I,X}_ *J*^2^_*i*_ν_*i, o*_τ_m_ are the self-consistent input mean and variance. Because all neurons receive statistically identical input, in absence of symmetry-breaking the firing rate distribution is spatially homogeneous, i.e., ν_*o, i*_ ≡ ν_*o*_ for all *i*. To assess simple stability of the homogeneous state to spatial perturbations, we need to perform linear perturbation analysis. The critical eigenvector of the resulting linear stability operator then determines the spatial pattern that develops.

We will show that the exact nature of the appropriate linearization depends critically on the input current regime, and derive the respective linearization in the limits of subthreshold mean input with fluctuation-driven spiking in section 3.1.1, and of dominant suprathreshold mean total input current μ[*R*(*I*_x_ + *I*_s_)] > θ in section 3.1.2. In the fluctuation-driven regime a quantitative approximation is obtained from the modulation of both mean and fluctuations of the synaptic input to the neurons, while in the mean-driven regime we recover the intuitive result that the system is governed by the noise-free input-output relation given by the *f*-*I*-curve of the individual neuron. In general, the full spiking dynamics will lie somewhere in between these two limiting cases, the case we call “intermediate.”

#### 3.1.1. Linearization of the input-output relation in the fluctuation-driven regime

We first discuss the case when the system is fluctuation-driven, i.e., the average input current is subthreshold (μ[*RI*] < θ) and spikes are induced by membrane-potential fluctuations.

To assess linear stability in this regime we need to derive a linear model that self-consistently describes the dynamics of the fluctuations *u*_*i*_(*t*) = 〈*s*_*i*_(*t*) − ν_*o*_〉 = ν_*i*_(*t*) − ν_*o*_ of the activity of neuron *i* around its mean firing rate or working point ν_*o*_. Treating the response of integrate-and-fire neurons by first order perturbation theory is a well-established method first introduced in Abbott and van Vreeswijk (1993) for the perfect integrator and in Amit and Brunel (1997); Brunel and Hakim (1999) for the leaky integrate-and-fire neuron.

The dynamics of *u*(*t*) can then be written in the form

(6) u(t)≃h(t)∗[u(t)+x(t)],

where *h*(*t*) is a linear filter and *x*(*t*) is a white noise imitating the spiking nature of the signal. In the Fourier-domain Equation (6) becomes the simple product *U*(ω) = *H*(ω)[*U*(ω) + *X*(ω)], where the capitalizations indicate the Fourier-transforms of *h*(*t*), *u*(*t*) and *x*(*t*). Pattern formation is typically a slow process and the transfer function *H*(ω) of leaky integrate-and-fire neurons is dominated by low-pass behavior^1^. In order to assess stability, we therefore estimate *H*(ω) in the low-frequency limit, i.e., $H(0)=\int_{0}^{\infty }h(t)\;dt$. With $\frac{d\mu_{o,i}}{ds_{j}}=\tau_{\text{m}}W_{ij}$ and $\frac{d\sigma_{o,i}^{2}}{ds_{j}}=\tau_{\text{m}}W_{ij}^{2}$ as well as $\frac{d\sigma_{o,i}^{2}}{ds_{j}}=2\sigma_{o,i}\frac{d\sigma_{o,i}}{ds_{j}}$, we obtain (see also Amit and Brunel, 1997; Tetzlaff et al., 2012; Helias et al., 2013 for details) the effective coupling matrix $\tilde{W}$ of the system as the local derivative of the input-output-curve given by Equation (5):

(7a) $${\tilde{W}}_{ij}(W_{ij})=\int_{0}^{\infty }h_{ij}(t)dt=\frac{\partial \nu_{o,i}}{\partial s_{j}}$$

(7b) $$=\frac{\partial \nu_{o,i}}{\partial \mu_{o,i}}\tau_{\text{m}}W_{ij}+\frac{\partial \nu_{o,i}}{\partial \sigma_{o,i}}\frac{\tau_{\text{m}}}{2\sigma_{o,i}}W_{ij}^{2}$$

(7c) $$\begin{array}{l} ={\left(\nu_{o}\tau_{\text{m}}\right)}^{2}\sqrt{\pi }\frac{W_{ij}}{\sigma_{o,i}}(f(y_{\theta ,i})\left(1+\frac{W_{ij}}{2\sigma_{o,i}}y_{\theta ,i}\right)\\ \;-f(y_{r,i})\left(1+\frac{W_{ij}}{2\sigma_{o,i}}y_{r,i}\right)), \end{array}$$

with

(8) $$f(y)=e^{y^{2}}(\text{erf}[y]+1),\;y_{\theta }=\frac{\theta -\mu_{o}}{\sigma_{o}},\text{ and }y_{r}=\frac{V_{\text{res}}-\mu_{o}}{\sigma_{o}}.$$

The effective coupling matrix $\tilde{W}$(*W*_*ij*_) is thus structurally identical to *W* given by Equation (2), with $\tilde{W}$_*ij*_ = $\tilde{W}$(*J*) if *j* is excitatory, and $\tilde{W}$_*ij*_ = $\tilde{W}$(−*gJ*) if *j* is inhibitory, yielding an effective relative inhibitory strength $\tilde{g}$ := |$\tilde{W}$(−*gJ*)/$\tilde{W}$(*J*)|. The zero frequency mode *U*(0) therefore fullfills the equation

(9) $$U(0)={\left[𝕀-\tilde{W}\right]}^{-1}X(0).$$

We expect linearity to break down if $\tilde{W}$ has any eigenvalue with real part larger than unity. The critical coupling strength *J*^fd^_*c*_ for which this is the case can be determined implicitly from Equation (7).

#### 3.1.2. Linearization in the low input current noise limit

Here, we discuss the linearization in the case that neurons are mean-driven, i.e., when the mean of the input current μ[*RI*] is suprathreshold, while the variance of the input is negligible [see also Amit and Tsodyks (1991) for analogous arguments]. In this case, the input-output-relation of the neuron becomes effectively linear in μ[*RI*] until it reaches saturation at ν_max_ = 1/τ_ref_ (Amit and Tsodyks, 1991; Salinas and Sejnowsky, 2000). This can be seen by considering the input-output relation of an individual neuron in response to a constant current, i.e., the *f*-*I*-curve

(10) $$\nu (I)={\left[\tau_{\text{ref}}-\tau_{\text{m}}\text{Log}\left[1-\frac{\theta }{RI}\right]\right]}^{-1}.$$

For *RI* » θ, i.e., $\frac{\theta }{RI}\ll 1$ and negligible τ_ref_, it holds (see Appendix A2 for details)

(11) $$\nu (I)≐\frac{RI}{\tau_{\text{m}}\theta }-\frac{1}{2\tau_{\text{m}}}.$$

**Figure 2:**
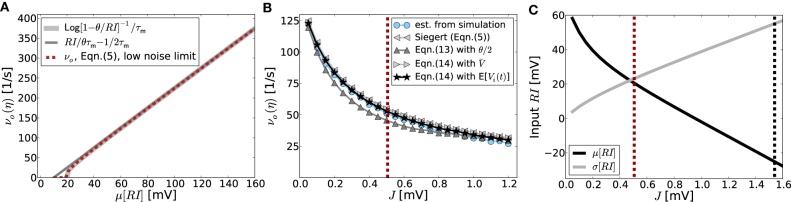
**(A)** Demonstrates that the input-output-relation of the LIF neuron Equation 5 indeed gets linear in the strongly mean-driven regime. The light gray line shows the *f*-*I*-curve in the noise-less case [Equation (10)], dark gray corresponds to its linear approximation Equation (11), while the red curve is the corresponding self-consistent rate given by Equation (5) in the low-noise limit σ[*RI*] → 0. **(B)** Shows the output rates as a function of network coupling strength *J* as they are actually obtained from network simulations averaged over 10 trials (blue dotted curve) vs. the prediction from Equation (5) (gray left-pointing triangles), as well as the linear model prediction Equation (13) with E[*V*(*t*)] = θ/2 (dark gray triangles). Also shown is the prediction from solving Equation (14) self-consistently with *V* = ∫^∞^_−∞_ *VP*(*V*) *dV* [with stationary membrane potential probability density function *P*(*V*) as derived in Brunel (2000); gray right-pointing triangles], as well as from using the estimated average membrane potentials E[*V*_*i*_(*t*)] obtained from simulations (black asterisks). The vertical red line represents the expected critical coupling strength *J*^md^_*c*_ for the mean-driven regime. The external drive is constant Poissonian noise (η = 3.5), while the local network coupling strength *J*, and hence μ[*RI*_s_] and σ[*RI*_s_], vary. In this setting the system undergoes a regime change from the mean-driven to the fluctuation-driven scenario. This is demonstrated in **(C)**, where the total mean and standard deviation of *RI* = *R*(*I*_x_ + *I*_s_) are shown as function of *J* in black and gray, respectively. The red dashed line corresponds to that in **(B)**, while the black dashed line indicates the critical *J*^fd^_*c*_ expected from Equation (9). The corresponding spike activity is shown for three exemplary cases in Figures 1G–I. Other parameters in **(B,C)** are *N* = 2500, κ = 250, θ = 20 mV, τ_m_ = 20 ms, τ_ref_ = 0.1 ms, and *g* = 6.

This linear-threshold-type relationship $\nu (I)={\left[\frac{RI}{\tau_{\text{m}}\theta }-\frac{1}{2\tau_{\text{m}}}\right]}_{+}$ fits that observed in simulations very well, cf. Figures 2A,B.

To derive a rate equation for the full spiking network dynamics, we first define the instantaneous rate of neuron *i* as

(12) $$\nu_{i}(t)={\text{E}}_{\Delta t}[s_{i}(t)]:={\text{lim}}_{\Delta t\to 0}\frac{1}{\Delta t}\int_{-\Delta t/2}^{\Delta t/2}\text{E}[s_{i}(t)]dt,$$

where E[.] is the average over realizations.

Substituting the spike trains by their respective firing rates, the input to neuron *i* takes the form *RI*_*i*_ = τ_m_ ∑_*j*_ *W*_*ij*_ ν_*j*_ + *RI*_x_. If we only consider networks with rates ν_*i*_ ≥ 0 and approximate the firing rate of neuron *i* by the linear expression (11), we arrive at the linear equation

(13) $$\begin{array}{l} \;\nu_{i}=-\frac{1}{2\tau_{\text{m}}}+\frac{1}{\theta \tau_{\text{m}}}\left(\tau_{\text{m}}\sum_{j}W_{ij}\nu_{j}+RI_{\text{x}}\right)\;\Leftrightarrow \\ \tau_{\text{m}}\nu ={\left[\theta 𝕀-W\right]}^{-1}(RI_{\text{x}}-\theta /2). \end{array}$$

Indeed, if τ_ref_ is negligible, Equation (1) can be rewritten as (Kriener et al., 2008)

(14) $$\tau_{\text{m}}\frac{dV_{i}(t)}{dt}=-V_{i}(t)-\tau_{\text{m}}\theta s_{i}(t)+\tau_{\text{m}}\sum_{j=1,i\neq j}^{N}W_{ij}s_{j}(t-d)+RI_{\text{x,i}}.$$

Equation (13) can then be derived analogously by temporal averaging, assuming stationarity *d* E_Δ*t*_[*V*(*t*)]/*dt* ≡ 0, i.e., E_Δ*t*_[*V*(*t*)] ≡ const, and thus E_Δ*t*_[ν(*t*)] = const = ν_*o*_. For high rates in response to large μ[*RI*] we expect E[*V*(*t*)] ≈ (*V*_thr_ − *V*_res_)/2 = θ/2, as the voltage passes through the interval between reset and threshold with approximately constant velocity, leading to

(15) $$0=-\theta /2-\tau_{\text{m}}\theta \nu_{i}(t)+\tau_{\text{m}}\sum_{j}W_{ij}\nu_{j}+RI_{\text{x,i}},$$

which is identical to (13). We note, that the self-consistent solution of Equation (15) yields the correct quantitative rates over a wide range of relative input magnitude, if instead of θ/2 the actual E[*V*_*i*_(*t*)]-values measured in simulations are inserted. If all neurons are identical and receive statistically identical input, this mean value can be obtained from ∀_*i*_ E[*V*_*i*_(*t*)] ≡ *V* := ∫^θ^_−∞_ *VP*(*V*) *dV*, where *P*(*V*) denotes the stationary membrane potential probability density function as e.g., derived in Brunel (2000). All output-rate predictions are compared to the outcome from simulations in Figure 2B. Aside from the prediction that assumes E[*V*(*t*)] = θ/2 (dark gray triangles) and underestimates the true rate, all predictions fit the simulation results (blue dots) very well. In particular, the Siegert equation (5) coincides perfectly with the linearized rate Equation (14) if we assume E[*V*(*t*)] = *V* (light gray triangles), while the best fit is obtained with Equation (14) and the actual measured E[*V*_*i*_(*t*)] (black asterisks).

The stability of the fixed point rate to small perturbations is determined by the largest real part of all eigenvalues of *W*/θ. If one eigenvalue λ_*c*_ has real part larger than unity, we expect the corresponding eigenvector *v*_*c*_ to grow exponentially, only limited by the non-linearities due to rate-rectification for small rates, and the saturation because of neuronal refractoriness for high rates. This determines the critical coupling strength

(16) $$J_{c}^{\text{md}}=\frac{1}{\text{Re}[\lambda_{c}]}$$

that in this limit is independent of the exact working point ν_*o*_ > 0. Even though for coupling strengths *J* > *J*^md^_*c*_ the rate instability can lead to pattern formation such that individual neurons fire at quite different rates, the population rate is still captured well by the firing rate predictions assuming homogeneous rates across neurons, see Figure 2B.

Finally, we note that for the constant external drive scenario considered here the mean and variance of the total input current vary with varying *J*. In particular, for increasing *J* the system undergoes a cross-over from the mean-driven to the fluctuation-driven regime, see Figure 2C.

### 3.2. Eigensystem of dale-conform translation-invariant ring networks

**Figure 3:**
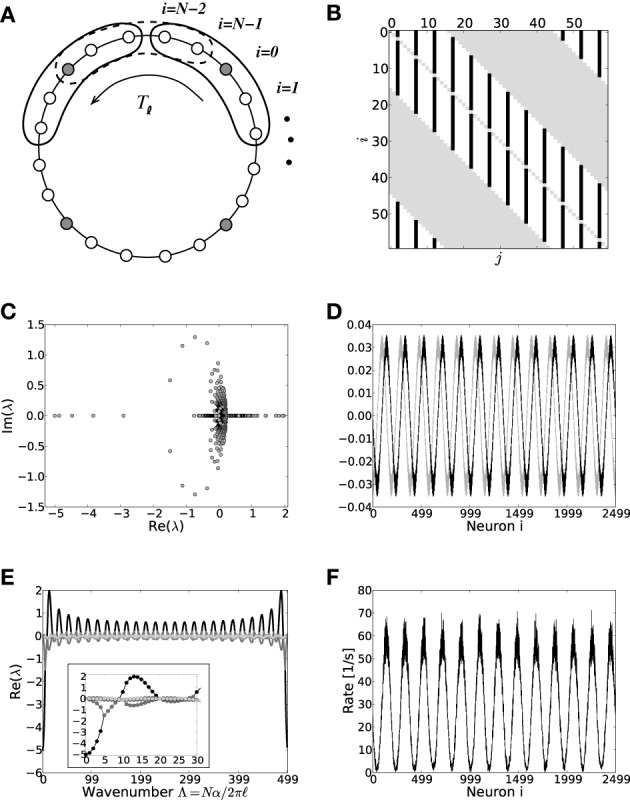
**(A)** Sketch of the neuron layout on the ring topology. Each neuron is connected to its κ nearest neighbors. A shift in neuron label by five yields the same ring as before and is formally expressed by the shift operator *T*_ℓ_ with ℓ = 5. **(B)** Shows the coupling matrix *W* of the ring: black squares depict inhibitory, white squares excitatory, and gray squares zero coupling from *j* to *i* (*N* = 60, κ = 30). **(C)** Eigenvalue spectrum of a ring matrix *W*/θ with *N* = 2500, κ = 250, *J* = 1 mV, and *g* = 6. **(D)** The two eigenvectors belonging to the twice degenerated eigenvalue from **(C)** with largest real part λ_*c*_. **(E)** The eigenvalues form five bands and are shown as a function of the wavenumber. The black curve depicts the band containing the critical (maximal) eigenvalue λ_*c*_ which here corresponds to wavenumber Λ_*c*_ = 13, so the two crictical eigenvectors have 13 major peaks (cf. **D,F**). **(F)** The rate as a function of the neuron index in a simulation of *N* = 2500 integrate-and-fire neurons with *J* = 1 mV and *I*_x_ = 750 pA. Note that the spectra (shown in **C**) look slightly different in the fluctuation-driven case, because the absolute amplitude of the entries |$\tilde{W}$_*ij*_| ≠ |*W*_*ij*_|, and also the balance between positive and negative entries $\tilde{g}$ ≠ *g*. The maximum of the dispersion relation and the eigenvectors are, however, the same.

In this section we will analytically derive the eigenvalue spectrum of the ring networks under consideration that yields the critical eigenvalue and thus the critical coupling strength *J*_*c*_ with respect to the two linearizations Equation (9) and Equation (13). As described in section 2 we consider ring networks of size *N* with regular connectivity, in that each neuron is connected to its κ nearest neighbors, κ/2 on each side of the neuron but not to itself. Inhibitory neurons shall be distributed periodically across the network as illustrated in Figure 3A, where the ratio between excitation and inhibition is 4 : 1 (β = 0.8). This is in line with the observed frequencies of excitatory and inhibitory cells in cortex (Schüz and Braitenberg, 1994). The ring can thus be divided into structurally identical elementary cells of four excitatory neurons and one inhibitory neuron. We choose κ as an integer multiple of the size ℓ of an elementary cell (here ℓ = 5, κ mod ℓ = 0), such that it moreover divides the total number of neurons *N* (*N* mod κ = 0). The resulting coupling matrix is sketched in Figure 3B, where black squares depict inhibitory weights, white depict excitatory weights and gray denotes the lack of a connection. In this way all neurons receive the exact same amount of inhibition and excitation, and the translation invariant mode (1, 1, ···, 1)ᵀ is an eigenvector of the coupling matrix with eigenvalue μ = κ*J*(β − *g*(1 − β)). To keep network activity balanced, inhibitory synapses are assumed to be *g*-times stronger, with *g* > 4, cf. Equation (2). Hence, the local network is inhibition dominated.

To compute the eigensystem of the *N*-dimensional system we make use of its symmetry properties. The coupling matrix commutes with the unitary operator that shifts all neuron indices by multiples of ℓ = 5. Hence, we can diagonalize both in the same eigenbasis and moreover reduce the problem to an effectively five-dimensional one as outlined in Appendix A3. Figure 3C shows the exemplary eigenvalue spectrum of in this case the rescaled coupling matrix *W*/θ of a network of size *N* = 2500 with absolute coupling strength *J* = 1 mV and relative strength of inhibition *g* = 6. Thus, the eigenvalue λ_*c*_ of *W*/θ with largest real part will exceed unity, if the amplitude of the absolute coupling strength *J* exceeds *J*^md^_*c*_ = 1/Re[λ_*c*_], cf. Equation (16), here *J*^md^_*c*_ ≈ 0.5.

The corresponding critical weight *J*^fd^_*c*_ for the fluctuation-driven case is obtained by the identical eigenvalue decomposition of $\tilde{W}$ as defined by Equation (7). Since $\tilde{W}$ depends non-trivially on the self-consistent working point ν_*o*_ of the system, the critical coupling strength *J*^fd^_*c*_ is the solution of

(17) Jcfd:=J such thatmaxi[Re[λi(W˜(νo,J))]]≡1,

where λ_*i*_, *i* ∈ {1, …, *N*} are the *N* eigenvalues of $\tilde{W}$. If *J* > *J*^md/fd^_*c*_ (depending on the regime), the spatially homogeneous rate distribution becomes unstable to perturbations in favor of the fastest growing eigenmode *v*_*c*_, which only depends on the symmetry properties of the matrix as long as *g* and $\tilde{g}$ are larger than four, and it is hence the same irrespective of the input regime, cf. Figures 1C,F,I. The spatial frequency of the emerging spatial pattern (see Figure 3), i.e., the number of maxima of the rate distribution along the ring, is thus given by the wavenumber Λ (see Appendix A3) that corresponds to the respective λ_*c*_ := max_*i*_[Re[λ_*i*_]]. We note, that the eigenvalue spectra in the fluctuation-driven case look slightly different from those in the mean-driven case (cf. Figure 3C) because of the in general different scaling $\tilde{g}$ ≠ *g* between positive and negative entries in $\tilde{W}$. The maximum of the dispersion relation determining Λ_*c*_ and the eigenvectors are, however, usually the same.

**Figure 4:**
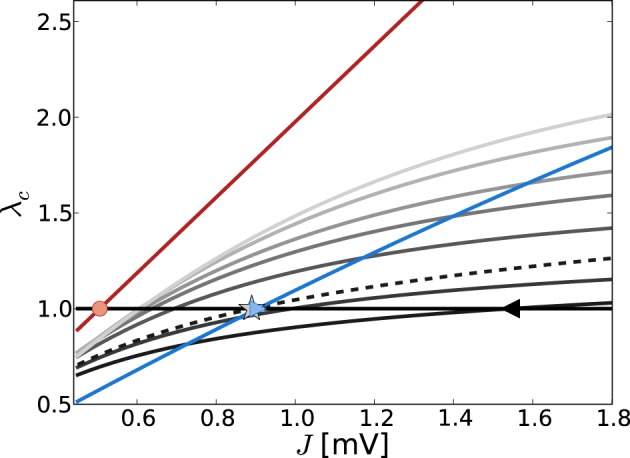
**Eigenvalue of *W*/θ (red) and $\tilde{W}$ (varying gray levels) with largest real part λ_*c*_ in dependence of *J* and external drive η (increasing η = {3.5, 5, 10, 15, 20, 30, 40} from dark to light gray)**. The eigenvalues of *W*/θ do not depend on η and thus the *J*_*c*_ value such that λ_*c*_ = 1 [indicated by red circle, cf. Equation (13)] holds uniquely for *J*^md^_*c*_ = 0.506. The gray lines show the dependence of λ_*c*_ of $\tilde{W}$ as obtained from Equation (7) with both contributions from *d*ν/*d*μ, *d*ν/*d*σ (solid lines) and with the *d*ν/*d*μ contribution only (dashed, here for clarity we just plot the curve for η = 3.5). The black triangle and the gray asterisk indicate the respective predictions of *J*^fd^_*c*_ = 1.54 and *J*^md, *d*ν/*d*μ^_*c*_ = 0.89 for η = 3.5. For comparison, the blue line depicts the *J*-dependence in the input scenario with fixed total input current mean and variance, i.e., μ[*RI*] = 5 mV and σ[*RI*] = 60 mV, independent of changes in *J*. In that case, the self-consistent population firing rate is expected to be constant and thus Equation (7) is of the form $\tilde{W}$ = *c*_1_*W*_*ij*_ + *c*_2_*W*^2^_*ij*_, with constants *c*_1_, *c*_2_. For large σ and *c*_1_ > *c*_2_ the curve is dominated by the linear weight dependence. For the parameters shown here, the expected *J*^fd^_*c*_ = 0.905 mV (cf. Figure 1).

Figure 4 shows the critical eigenvalue λ_*c*_ as a function of *J* for the linearizations Equation (9) (gray lines) and Equation (13) (red line) in the constant external drive regime, parameterized by η. The critical eigenvalue *J*_*c*_ is given by the intersection with the horizontal line at one. As was pointed out in section 3.1.2, in the noiseless approximation Equation (13) the eigenvalues of the linear operator do not depend on the working point, and thus the critical weight *J*^md^_*c*_ is unique for all η such that ν_*o*_ > 0 (here, critical *J*^md^_*c*_ = 0.506 indicated by red circle).

For the linearization Equation (9), and taking into account both μ and σ in Equation (7b) (solid lines), the critical weight *J*^fd^_*c*_ is strongly dependent on the value of η = {3.5, 5, 10, 15, 20, 30, 40} (increasing from dark to light gray), and never comes close to the prediction of the noiseless linearization *J*^md^_*c*_. In particular, for larger η *J*^fd^_*c*_ increases again.

If we neglect the σ-dependence in Equation (7b), and just take into account *d*ν_*o*_/*d*μ_*o*_ = *d*ν^md^_*o*_/*dRI* = 1/τ_m_θ as given by Equation (11), Equation (7) is linear in *W*_*ij*_ and we recover the linear noiseless approximation Equation (13) for Equation (9). We might thus expect that Equation (9) in general will give a *J*^fd^_*c*_-prediction close to *J*^md^_*c*_ if we neglect the σ-dependent quadratic term in Equation (7b). Although the prediction of this *J*^fd|*d*ν_*o*_/*d*μ_*o*_^_*c*_ becomes smaller (cf. black triangle for *J*^fd^_*c*_ = 1.54 mV vs. the gray asterisk for *J*^fd|*d*ν_*o*_/*d*μ_*o*_^_*c*_ = 0.89 mV for η = 3.5) it never becomes as small as *J*^md^_*c*_. This is because every change in μ_*o*_ will also influence σ_*o*_ via its impact on ν_*o*_, and thus *d*ν_*o*_(μ_*o*_, σ_*o*_)/*d*μ_*o*_ ≠ *d*ν^md^_*o*_(*RI*)/*dRI*.

We note that the observation *J*^fd^_*c*_ > *J*^fd,*d*ν_*o*_/*d*μ_*o*_^_*c*_ is explained by the fact that $\tilde{W}$(*W*_*ij*_)|_*d*ν_*o*_/*d*μ_*o*__ is linear in *W*_*ij*_, and thus the ratio $\tilde{g}$ = |$\tilde{W}$(−*gJ*)/$\tilde{W}$(*J*)| = *g*, while for the full Equation (7), $\tilde{W}$(*W*_*ij*_) is quadratic in *W*_*ij*_, yielding a $\tilde{g}$ < *g*. Evaluating Equation (A20) in Appendix A3 shows that the critical eigenvalue λ_*c*_ in both linearizations is a linearly increasing function of *g*, $\tilde{g}$, respectively, and *J*_*c*_ is thus decreasing as a function of *g*, $\tilde{g}$.

For comparison, Figure 4 also shows the *J*-dependence of the real part of the critical eigenvalue λ_*c*_ in the strongly fluctuation-driven regime (μ[*RI*] = 5 mV, σ[*RI*] = 60 mV, blue line), with the critical *J*_*c*_ indicated by the crossing of the unity-line (blue triangle).

### 3.3. Linear stability in dependence of the input current regime

We will now compare the general performance of the linearizations Equations (9) and (13) and in particular the resulting predictions of the critical coupling strength *J*_*c*_ with the actual onset of pattern formation as observed in simulations. Figure 1 demonstrates how the identical ring network structure undergoes pattern formation at very different values of network coupling strength *J* depending on the input current regime. In particular, pattern formation sets in later if the system is in a fluctuation-driven regime. This can be understood in the two limit-cases that were analyzed in sections 3.1.1 and 3.1.2.

But what determines pattern formation onset in intermediate cases that comprise the most interesting and relevant cases? As we saw in section 3.1.2, the network will even undergo a crossover from the mean-driven to a more fluctuation-driven regime, if the external input is kept constant and not adapted to counterbalance the changes in recurrent input structure for changing *J*, see Figure 2C. Also, due to the spiking nature of the activity, even in a strongly mean-driven scenario there will always be a considerable amount of variance which might change the onset of pattern formation with respect to *J*^md^_*c*_.

**Figure 5:**
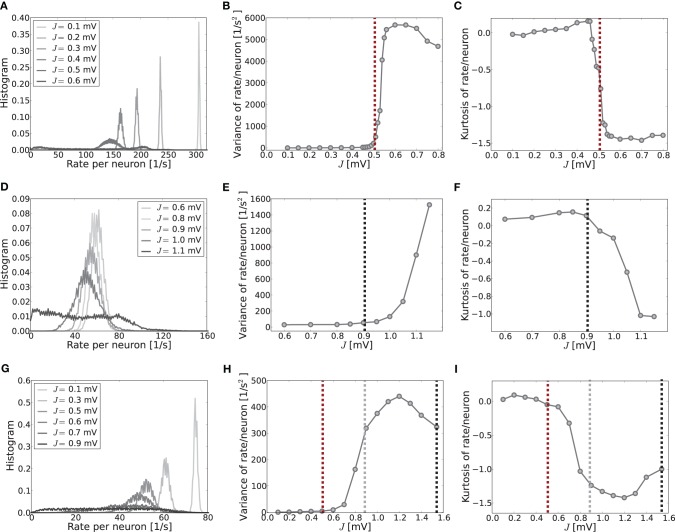
**The histograms of rates, variance and kurtosis for various weights *J* in a network of *N* = 2500, κ = 250, and *g* = 6 averaged over 10 trials**. **(A–C)** Shows the mean-driven case (*I*_x_ = 2500 pA delivered as Poisson noise with external synaptic strength *J*_x_ = 0.1 mV), **(D–F)** the fluctuation-driven case (Poisson noise adapted s. t. μ[*RI*] = 5 mV and σ[*RI*] = 60 mV) and **(G–I)** an intermediate case, where the external input was Poisson noise (current-amplitude 875 pA, *J*_x_ = 0.1 mV). For subcritical *J* < *J*_*c*_ the distributions are approximately Gaussian with small variance and kurtosis close to zero. For supracritical weight the distributions become broader and platykurtic, indicated by the negative kurtosis. The vertical dashed lines in **(B,C)** and **(E,F)** mark the respective critical weights, i.e., *J*_*c*_ = 0.506 mV [red, using Equation (13)] for the mean-driven, and *J*_*c*_ = 0.905 mV for the strongly fluctuation-driven case [black, using Equation (9)]. The dashed vertical lines in **(H,I)** mark the respective predictions for the critical weight by Equation (13) (red, independent of η), and by Equation (9) with only the contribution of ∂ν/∂μ (gray) and both linear and quadratic contributions ∂ν/∂μ, ∂ν/∂σ (black) for the estimation of the effective coupling strengths, cf. Equation (7b). The increase in the kurtosis and decrease in the variance for very large *J* in **(B,C)** and **(H,I)** is due to the rectification of rates that leads to increasing mass at zero. Note that sampling is denser around the expected *J*_*c*_-value in the first two rows.

To give more insight into the actual onset of pattern formation, useful reduced measures are the variance and kurtosis of the distribution of firing rates over neurons, cf. Figure 5. Figures 5A–C show the histogram of rates, and the variance and kurtosis of these histograms for various *J*-values in the mean-driven regime averaged over 10 trials each. Figures 5D–F show the same for the fluctuation-driven case and Figures 5G–I for the intermediate case, all for the same parameters as in Figure 1.

When the system is still well in the linear regime *J* < *J*_*c*_ the distribution is approximately Gaussian with small variance and a kurtosis close to zero (cf. Figures 5A,D,G, light gray curves), while once the critical weight *J*_*c*_ is surpassed the variance increases strongly (cf. Figures 5B,E,H) and the kurtosis becomes negative (platycurtic), indicating the broadening of the distribution due to the inhomogeneous rate per neuron (cf. Figures 5C,F,G and 5A,D,G, dark gray curves). The respective critical weights are indicated by vertical dashed lines in Figures 5B,E,H and C,F,I for visual guidance (red: *J*^md^_*c*_, black: *J*^fd^_*c*_, gray: *J*^fd|*d*ν_*o*_/*d*μ_*o*_^_*c*_).

The onset of pattern formation in the strongly fluctuation-driven regime at *J*^fd^_*c*_ is more shallow than in the mean-driven regime at *J*^md^_*c*_. This might be due to deviations of the input spike statistics from the asynchronous-irregular activity assumption underlying the linear response derivation of *J*^fd^_*c*_: the standard-deviation of the input is extremely large at σ = 60 mV. This implies that the membrane-potential will be hyperpolarized for long times, while at other times it is highly depolarized, and several spikes are emitted in short succession. This typically leads to higher coefficients of variation, i.e., more irregular firing than expected for Poisson spike statistics [see also Brunel (2000) and supplementary material section S1]. Also, even in the highly fluctuation-driven regime there may be residual correlations between spike trains. Lastly, the increased network coupling strength might lead to deviations from the Gaussian white noise approximation of input currents underlying Equation (5) and the linear response theory yielding Equation (7) (see supplementary material section S1 for an analysis). How such deviations of the input statistics can indeed change the spike response behavior of LIF neurons was studied, e.g., in Moreno et al. (2002); Renart et al. (2007); Moreno-Bote et al. (2008); Helias et al. (2010).

However, another contributing factor, and more likely the reason for the shallow transition at *J*^fd^_*c*_, is the degeneracy of the maximal eigenvalue, see Figures 3D,E. In the fluctuation-driven regime the system is subject to strong perpetual perturbations that lead to a switching between the two dominant eigenvectors depicted in Figure 3D in the transition regime *J*^fd^_*c*_ ± Δ*J*. Indeed, as can be seen from Figure 1E, the periodic pattern is already clearly distinguishable at *J*^fd^_*c*_ over several hundred milliseconds, but the average activity depicted in the histogram is still quite flat, yielding smaller variance and kurtosis of the respective rate distribution.

Finally, Figures 5D–F demonstrate clearly how pattern formation occurs for intermediate *J*-values *J*^md^_*c*_ ≲ *J*^inter^_*c*_ < *J*^fd^_*c*_ if the system is neither clearly in the mean- nor strongly fluctuation-driven regime, and even changes from one to the other regime with increasing coupling strength, cf. Figure 2C. As we demonstrate in the supplementary material section S1, most input and network settings have pattern formation onsets that usually agree much better with the *J*^md^_*c*_ prediction than with *J*^fd^_*c*_. This is a very consistent finding, even in cases where neurons are well in the fluctuation-driven regime in terms of subthreshold mean and pronounced input variance.

As we discuss in the supplementary material section S1 the reason for this finding lies in an asymmetry between the excitatory and inhibitory compound input current statistics. The excitatory subpopulation tends to be synchronized, even in the presence of strong balanced external noise, while inhibition actively decorrelates itself. The explanation for this effect lies in the local connectivity of ring networks, leading to a population-specific pattern of correlations already below the critical coupling: excess synchrony of the excitatory population will reinforce further spiking, while spikes from the inhibitory population decreases the instantaneous firing probability [see also Helias et al. (2013) for a related discussion of this effect in the framework of balanced random networks]. Near synchrony is thereby effectively enhanced in the excitatory population and suppressed in the inhibitory one. Volleys of excitatory input spikes act like compound pulses with large amplitude (on the order of up to θ) in an otherwise balanced asynchronous background activity. At these amplitudes the effective gain Equation (7) derived from linear response theory becomes linear in *J* with a slope proportional to 1/θ, i.e., the slope of the linear model Equation (11), see supplementary material section S1. This explains the fact that Equation (13) has better predictive power also in the intermediate or—comparably weakly—fluctuation-driven scenarios. Moreover, the fluctuation-driven prediction only becomes valid if the additional external noise sufficiently dilutes residual synchrony, such as it is the case for μ[*RI*] = 5 mV and σ[*RI*] = 60 mV.

### 3.4. Coarse-grained ring network

In section 3.2 we saw that the full system can effectively be reduced to a five-dimensional system. However, the computation of eigensystems (cf. Appendix A3) is still quite involved. In this section we study how well a further simplified system that is formally identical to the well-known Ermentrout-Cowan networks [Ermentrout and Cowan (1979a,b, 1980) and supplementary material section S2] predicts the dynamics of the full network.

**Figure 6:**
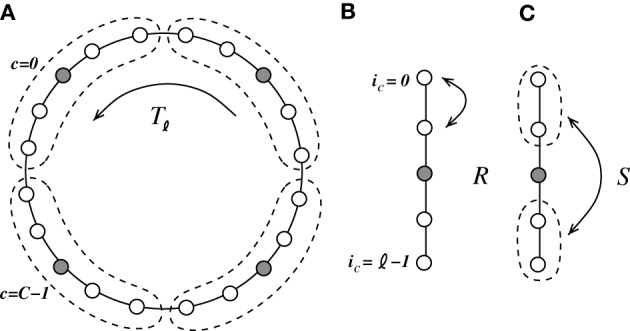
**We coarse-grain the system by introduction of cells containing four excitatory neurons and one inhibitory neuron [indicated in (A) by the dashed line]**. The coarse-grained system is invariant to three symmetry operations. **(A)** *T*_ℓ_ is a translation of the cell by ℓ = 5, **(B)** *R* is the mirror symmetry of two neighboring excitatory neurons within each cell, and **(C)** *S* is the mirror symmetry of the two pairs of neighboring excitatory neurons within each cell.

We coarse-grain the system and combine groups of ℓ = *N*/*N*_*I*_ neurons, such that they contain four excitatory and one inhibitory neuron (ℓ needs to be odd in the following), as indicated in Figure 6. The neurons *i*_*c*_ ∈ {0, …, ℓ − 1} within each cell *c* ∈ {0, …, *C* − 1}, *C* = *N*/ℓ, are connected to every other neuron within their cell *c* but not to themselves. Moreover, all neurons within one local cell *c* are connected to all neurons within the *K* < *C*/2 neighboring cells to the left and to the right, i.e., {(*c* − *K*) mod *C*, …, (*c* − 1) mod *C*, (*c* + 1) mod *C*, …, (*c* + *K*) mod *C*}. The computation of the eigensystem is outlined in Appendix A4. Due to the two mirror symmetries corresponding to exchange of neurons within the unit cell (with corresponding eigenvalues *r, s* ∈ {−1, 1}) and the translational symmetry with the eigenvalue determined by the wavenumber α, the system can be reduced to effectively two dimensions.

#### 3.4.1. Coarse-grained network dynamics

In the coarse-grained system we only need knowledge about two elements of the ring, one excitatory and one inhibitory neuron within the same cell (arbitrarily chosen as *v*_0_, *v*_(ℓ − 1)/2_ in *c* = 0, without loss of generality). For a fixed set of eigenvalues *r, s*, α, the activities of all remaining neurons are then uniquely determined. The homogeneous mode ν_*o*_(*t*) couples to the symmetric subspace *r, s* = 1 only and we can write ν_*o*_(*t*) thus as a linear combination of the two eigenvectors *v*^I^_α_0__ and *v*^E^_1,1,α_0__, where α_0_ = 0 (cf. Appendix A4).

To analyze the stability of this mode with respect to spatial perturbations we write the population activity as a linear combination of eigenvectors with non-zero wavenumbers α_*i*_, *v*^E^_1,1,α_*i*__, *v*^I^_α_*i*__, such that

(18) $$\nu (t)=\sum_{i=0}^{C-1}a_{i}(t)\left(v_{1,1,\alpha_{i}}^{\text{E}}+v_{\alpha_{i}}^{\text{I}}\right).$$

Then, for all neurons *n* ∈ {0, …, *N* − 1} the input, (*W* ν(*t*)) [*n*], in the reduced system becomes

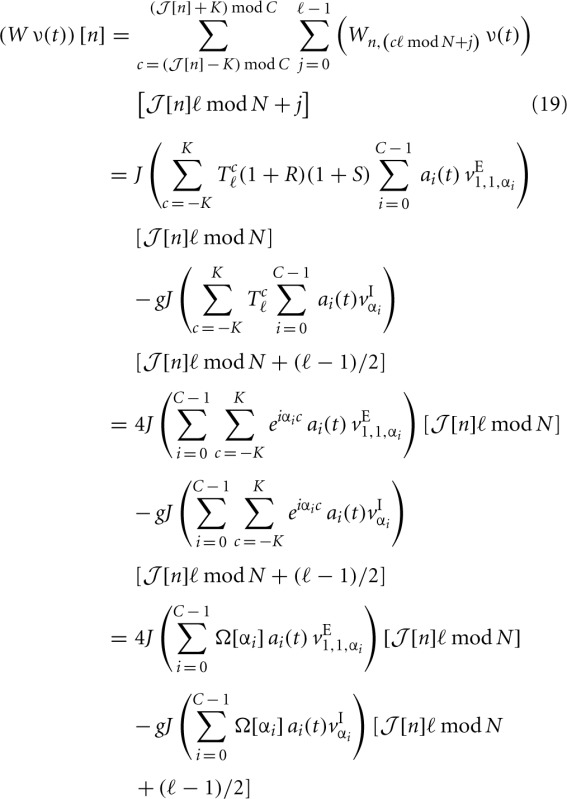



with 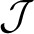
[*n*] := ⌊*n*/ℓ⌋. In the first identity we made use of the operators *R, S* and *T*_ℓ_ to express the input-connectivity of the network. The second identity follows from the linearity of the sum over eigenmodes and the respective eigenvalues of the operators *R, S* and *T*_ℓ_. We excluded synaptic self-coupling, so we need to subtract that input of the cell to itself, i.e.

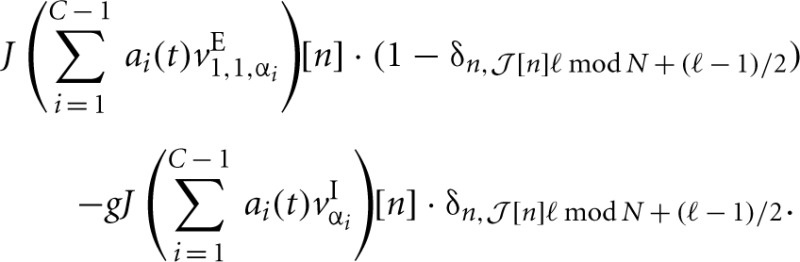



Leaving out one inhibitory input if the receiving neuron is inhibitory, and one excitatory input if the receiver is excitatory leads to an asymmetry in the total input of excitatory and inhibitory neurons. This implies that the spatially homogeneous mode is not an eigenmode of the system anymore. To compensate for this the weights are rescaled such that the input per neuron equals that in the full (non-coarse-grained) system, i.e., to μ = κ*J*(β − *g*(1 − β)). Hence,

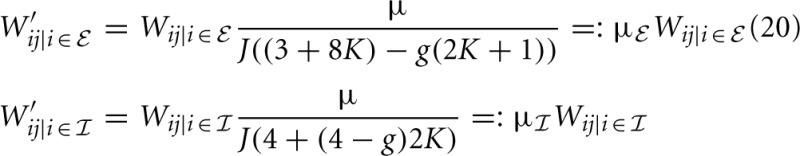



Note, that if we do not exclude synaptic self-coupling in the coarse-grained system the eigensystem looks dramatically different from that of the full system. In particular the eigenvalues are nearly fully real and the critical eigenvalue is much smaller implying a higher critical weight.

In the homogeneous mode the input to each neuron *n* ∈ {0, …, *N* − 1} is completely identical, all neurons fire at the same rate ν_*o*_ given by the self-consistent solution

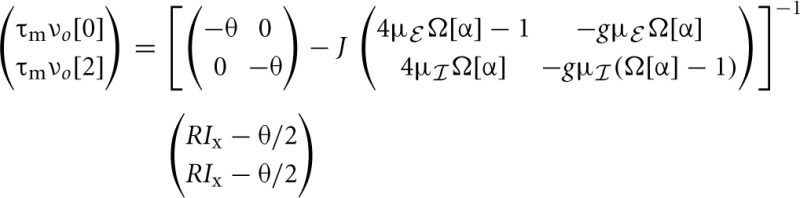



where $\Omega (\alpha ):=\frac{\text{sin}(\alpha (K+1/2))}{\text{sin}(\alpha /2)}$. The eigenvalues of the reduced rescaled coupling matrix

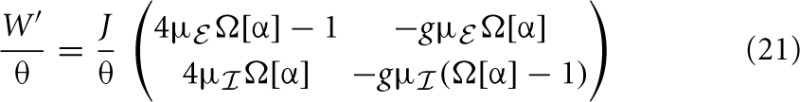



then determine for which parameters *g, J* the spatially homogeneous state becomes unstable and which wavenumber Λ_*i*_(α_*i*_) will grow fastest and define the spatial pattern. The critical eigenvalue for the coarse-grained system is thus given by the eigenvalue of $\frac{W^{\prime }}{\theta }$ with largest real part that will cross unity from below first, i.e.

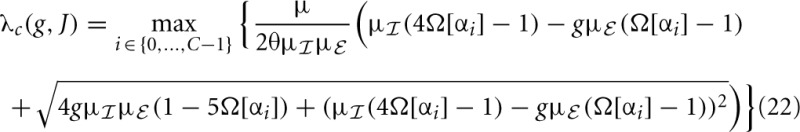



In the fluctuation-driven regime *J* and *g* in Equation (21) need to be substituted by $\tilde{J}$: = $\tilde{W}$(*J*) and $\tilde{g}$, such that the critical eigenvalue is given by λ^fd^_*c*_($\tilde{g}$,$\tilde{J}$).

**Figure 7:**
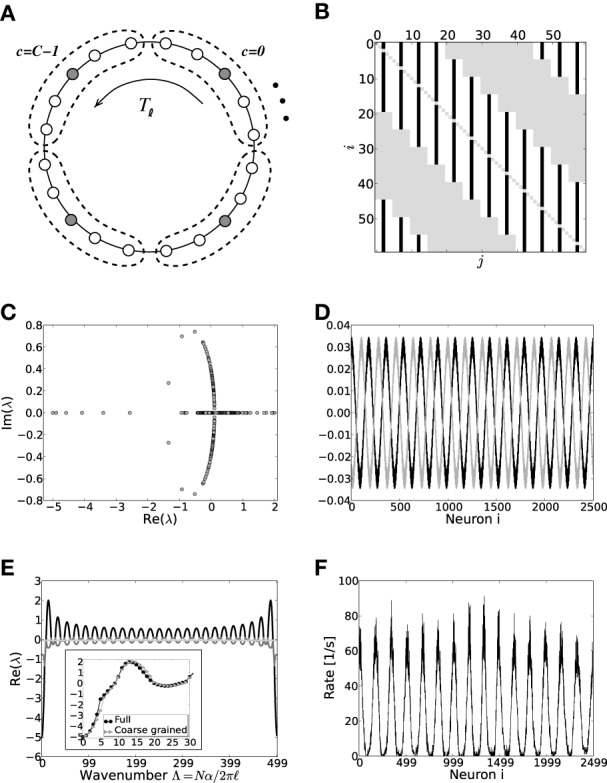
**(A)** Sketch of the layout of the coarse-grained system. Each neuron is connected to the *L* − 1 other cells within its cell and to all neurons in its 2*K* nearest cell neighbors. A shift in neuron label by five yields the same ring as before and is formally expressed by the shift operator *T*_ℓ_ with ℓ = 5. **(B)** Shows the coupling matrix *W* of the ring: black squares depict inhibitory, white squares excitatory, and gray squares zero coupling from *j* to *i* (*N* = 60, κ = 30). **(C)** Eigenvalue spectrum of a scaled ring matrix *W*/θ with *N* = 2500, κ = 250, and *g* = 6. **(D)** The two eigenvectors belonging to the twice degenerated eigenvalue from **(C)** with largest real part, λ^md^_*c*_. **(E)** Five bands of eigenvalues as a function of the wavenumber. The black line depicts the band containing λ^md^_*c*_. The inset shows the critical bands of the system in section 3.2 (black circles, cf. also Figure 3) vs. the critical band in the coarse-grained system (gray triangles). The critical wavenumber differs by one. **(F)** The corresponding rate per neuron in a simulation of *N* = 2500 integrate-and-fire neurons with *J* = 1 mV and *I*_x_ = 750 pA.

On a mesoscopic level the coarse-grained and full system give rise to very similar activity patterns—differences become apparent in the fine-scale features, e.g., the full analysis of a network of 2500 neurons with 10% connectivity and *g* = 6 predicts destabilization of wavenumber 14 in both mean- and fluctuation-driven regime, while the coarse-grained model predicts 13, see also Figure 7. We note that the coarse-grained model presented in this section is formally identical to the linearization derived in Ermentrout and Cowan (1980) in the context of a neural field ring model if one assumes a boxcar-footprint. We summarize the respective derivation in the supplementary material section S2.

### 3.5. Sensitivity to randomness

To conclude, we study how robust the findings of the previous sections are to effects of randomness in either structure or weight distribution. Though we again only show the eigenvalue spectra for the synaptic coupling matrix *W*/θ, that is the relevant linear operator in the mean-driven case, all results map straight-forwardly to the fluctuation-driven regime with appropriate rescaling of *J* and *g*.

#### 3.5.1. Small-world networks: structural noise

**Figure 8:**
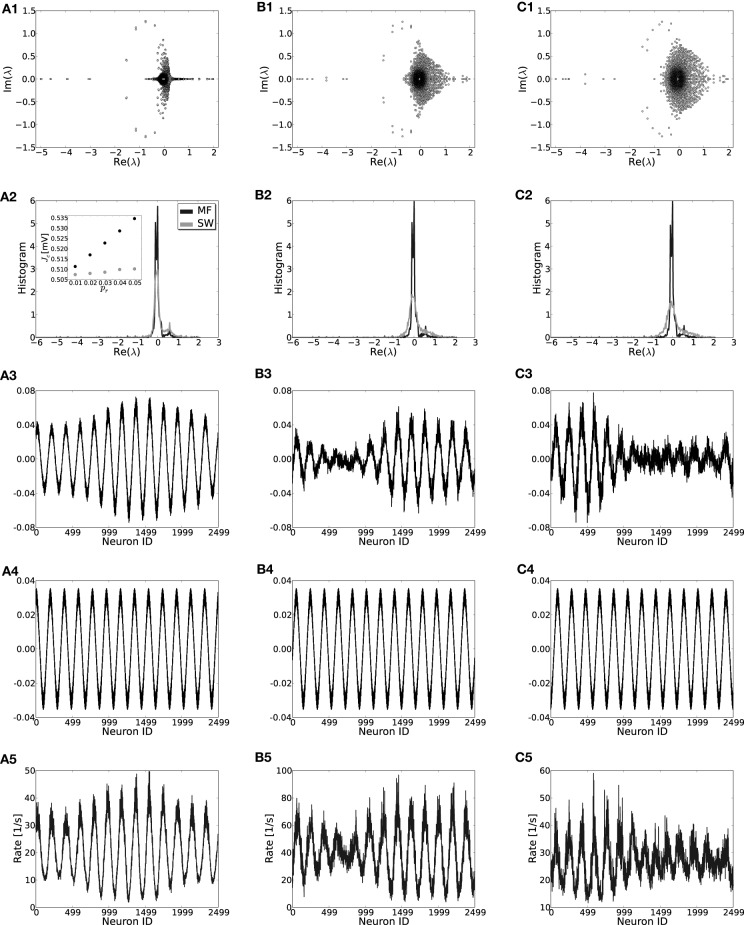
**Effect of rewiring of edges on the eigenvalue distributions (row 1), the mean-field density of the real parts of eigenvalues (row 2), the crictical eigenvectors of the specific realization (row 3) and the mean-field system (row 4) and the rate per neuron (row 5) in small-world networks for three different rewiring probabilities *p*_*r*_ = 0.01, 0.03, 0.05 in (A,B,C), respectively (cf. Watts and Strogatz, 1998; Kriener et al., 2009)**. While in **(A3,B3)** it is the dominant eigenmode *v*_*c*_ that correlates strongest with the actual rate distribution [**(A5,B5)**, Pearson correlation coefficients of *c* = 0.967 and *c* = 0.945, respectively], in **(C3)** the linear combination of the two leading eigenvectors correlates best with the actual rates [**(C5)**, *v*_*c*_ has *c* = 0.68 while the combination of the two leading modes gives *c* = 0.91]. The inset in **(A2)** shows the estimate for the critical weight *J*_*c*_ as a function of *p*_*r*_ in the mean field model (MF, black) and as an average of 50 network realizations (SW, gray). Parameters: *N* = 2500, *J* = 1 mV and *I*_x_ = 750 pA. During rewiring the number of excitatory and inhibitory inputs was kept constant.

First, we will study the effect of the introduction of structural randomness by rewiring individual connections with probability *p*_*r*_ [for details see Kriener et al. (2009)] such that the input composition from the network to every neuron stays constant at μ. The random rewiring procedure means that each realization of a network will be different and moreover lacks invariance to *T*_ℓ_ such that the eigensystems of individual coupling matrices need to be computed numerically. For low *p*_*r*_ the rate model for the translation-invariant network still gives very good results and the patterns that form for large *J* are indeed strongly correlated with the critical eigenvector *v*_*c*_, see Figure 8. For higher *p*_*r*_ predictions become worse and more than one vector can contribute to the pattern (e.g., Figures 8C2,C3). For low rewiring probability *p*_*r*_ < 0.02 we can still analytically estimate the critical eigenvalue by employing a mean field model (Grabow et al., 2012) for small-world networks in which we rewire “on average,” cf. Appendix A5 and Figures 8A2–C2,A4–C4. Hence, also for small-world networks of excitatory and inhibitory spiking neurons, rates and linear stability can be estimated from the translation-invariant network rate model if *p*_*r*_ is small. The results of the mean-field model are slightly worse than for unweighted networks (cf. Grabow et al., 2012) because of the two qualitatively different types of edges in the networks considered here (excitatory and inhibitory). For the small number of edges κ we assume in Figure 8 it can occur that for very small *p*_*r*_ no inhibitory edges are rewired at all, while the mean-field model still reassigns inhibitory connection density. We find the mean field to work better for denser or larger networks, and if the excitatory and inhibitory input per neuron is kept the same during rewiring (not shown).

If the constant input per neuron constraint is dropped, the homogeneous mode (1, …, 1)ᵀ is not an eigenmode anymore and also the prediction of stability fails in large parts. On the one hand there are activity inhomogeneities forming out simply due to the fact that some areas on the ring will by chance get more or less net inhibitory input, and hence some neuronal subpopulation will be driven to zero rates, while others—lacking net inhibitory input from these rectified neurons—will have very high rates. Similar effects arise when the distribution of inhibitory neurons is not periodic anymore, but, e.g., distributed randomly along the ring.

#### 3.5.2. Ring networks not conform with Dale's principle

**Figure 9:**
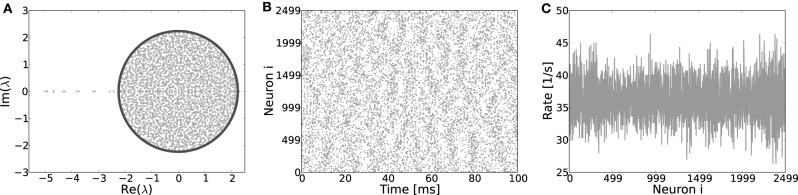
**(A)** Eigenvalue distribution in the complex plane of a ring network with random assignment of weights, irrespective of the identity of a neuron, hence each existing synapse has weight *J* with probability β and −*gJ* with probability (1 − β). These ring coupling matrices have eigenvalue spectra very akin to those of corresponding random networks and most of the eigenvalues adhere to the circle law prediction (Girko, 1984) for random networks $r=\sqrt{Np(\beta +g^{2}(1-\beta ))-\mu^{2}/N}$ (dark line). Only a few singular eigenvalues on the left of the center indicate the underlying ring topology. The eigenvectors of such “hybrid” ring matrices have no apparent structure and no pattern formation occurs when the system becomes supracritical, as clearly visible from the spiking activity **(B)** and the rates per neuron **(C)**. Here, *J*_*c*_ ≈ 0.44 mV, simulation parameters *J* = 0.6, *g* = 6 and *N* = 2500.

Finally, if Dale's principle—i.e., the biological fact that each neuron can only either depolarize or hyperpolarize all its postsynaptic targets, but never both at the same time—is violated, both the eigensystem (Figure 9A), as well as the spiking dynamics looks akin to that of a random network, even if the underlying topology is a ring, cf. Figures 9B,C. Hence, the identity of neurons in terms of being excitatory or inhibitory is a necessary condition for the formation of structured patterns in the case of identical footprints for inhibition and excitation considered here.

Even though in all cases pattern formation is either strongly or completely impaired, the prediction Equation (14) of individual rates in the low coupling regime is excellent, also in inhomogeneous networks, where different neurons receive very different inputs, given the individual mean membrane potentials 〈*V*_*i*_(*t*)〉_*t*_ are used in Equation (15).

## 4. Discussion

In this paper we analyzed the dynamics of pattern formation in networks of excitatory and inhibitory spiking neurons that are embedded on a ring architecture. In particular, we studied the dependence of the underlying bifurcation on the input current statistics and structural noise, and derived a coarse-grained system that is formally analogous to the classical Ermentrout-Cowan networks. To conclude, we will summarize and discuss our findings.

### 4.1. Impact of input current statistics

The very nature of spiking neuron networks leads to considerable input current fluctuations, and hence in general both mean μ[*RI*] and standard deviation σ[*RI*] of the input will shape the output rate of the neuron. For leaky integrate-and-fire neurons the output rate as a function of μ[*RI*] and σ[*RI*], assuming stationary Poisson-like uncorrelated input spike trains and weak synaptic coupling *J*, is given by the Siegert equation (5) (Amit and Tsodyks, 1991; Amit and Brunel, 1997). Thus, in order to analyze the linear stability of this self-consistent solution, in general the derivatives with respect to both μ[*RI*] and σ[*RI*] need to be taken into account, cf. Equation (7).

We analyzed two different ways to drive the networks: in the first all neurons in the network were driven with the same excitatory Poisson-noise ν_X_, independent on the recurrent coupling strength *J*. In this case the total input *RI* the neurons receive, i.e., the external input *RI*_x_ together with the recurrent input *RI*_s_ from the network, changes with increasing *J*, such that the mean μ[*RI*] decreases, while the standard deviation σ[*RI*] increases.

In the second input scenario, we corrected for the change in recurrent input contributions with changing *J* by administering compensating inhibitory and excitatory external Poisson input ν_Ix_, ν_Ex_. Thus, μ[*RI*] and σ[*RI*], and hence also ν_*o*_ are approximately constant with increasing *J*.

We find that in the first input scenario the critical *J*_*c*_ as observed in simulations deviates strongly from the one predicted by Equations (7),(9), which are derived from the self-consistent solution Equation (5) by linear response theory. In particular, for the mean-driven regime, where μ[*RI*] > *V*_thr_, *J*^md^_*c*_ becomes basically independent of the rate ν_*o*_ as well as of σ[*RI*], irrespective of its yet considerable magnitude. Instead, a very simple linearization derived from the noiseless *f*-*I*-curve Equation (10) applies. The *J*^md^_*c*_ derived from this linearization is always considerably smaller than the prediction by Equations (7), (9). For intermediate cases where neurons are neither mean- nor strongly fluctuation-driven, the critical coupling strength lies in between the two predictions, and often closer to *J*^md^_*c*_.

Possible reasons for the failure of the prediction of Equations (7), (9) in the constant external drive scenario (parameterized by η, cf. section 2) are the break-down of the weak-coupling assumption, as well as the uncorrelated Poisson assumption for the input spike trains in the recurrent contribution. In line with earlier findings (Tetzlaff et al., 2012), we could not identify deviations with respect to coupling strength, however, we observed clear deviations from the Poisson assumption.

If spiking is Poissonian we expect the interspike intervals to be distributed exponentially, and the coefficient of variation (CV) of the interspike intervals (ISI) to be unity. A smaller CV indicates more regular, a larger one more irregular spiking. As can be seen from Figures 1A–C and 1G–I, activity is spatio-temporally structured, with locally clustered spiking that can induce significant pairwise correlations, and low coefficients of variation of the interspike intervals for small *J* (cf. also supplementary material section S1, Figures S1B,C). In effect, these deviations from uncorrelated Poisson spiking lead to deviations from the Gaussian white noise approximation underlying the derivation of Equations (5) and (9), especially for increasing *J*.

We observe that in particular the excitatory subpopulation tends to synchronize because of the highly recurrent local structure and positive feedback, while the inhibitory population actively desynchronizes itself due to negative feedback (see supplementary material section S1, Figures S3, S4, and Helias et al. (2013) for a related discussion). Thus, high amplitude volleys of excitatory input in a balanced background comprised of asynchronous inhibition and external Poisson-drive move the network in a regime that is better captured by the mean-driven low-noise approximation Equation (11).

Activity in the strongly fluctuation-driven regime, on the other hand, is much more asynchronous and irregular by construction, cf. Figures 1D–F with CV[ISI] of unity and larger (see supplementary material, section S1, Figures S1B,C). When in this scenario σ[*RI*] is very large, while μ[*RI*] is sufficiently subthreshold, the prediction for the critical coupling strength *J*^fd^_*c*_ from Equations (7), (9) for linearity to break down indeed appears to be correct, or even seems to underestimate the actual onset of pattern formation.

This shallow transition around *J*^fd^_*c*_ can be explained by slow noise-induced transitions between the two degenerate eigenvectors which grow quickest once pattern formation takes place. However, the fact that the CV is larger than unity will also lead to deviations from the prediction. In a series of papers Moreno et al. (2002); Renart et al. (2007) and Moreno-Bote et al. (2008) studied the impact of deviations of the Poisson-assumption of input spike trains on the firing rates of neurons. They studied the effect of positive and negative spike train auto- and cross-correlations parametrized by the Fano-factor *F* = σ^2^[counts]/μ[counts] of spike counts on the output firing rate of current-based LIF neurons in both the fluctuation- and the mean-driven regime. For renewal processes the CV of interspike intervals is directly related to the Fano-factor of counts by *F* = CV^2^ (Cox, 1962). In Moreno et al. (2002); Renart et al. (2007); Moreno-Bote et al. (2008) it was shown that in the fluctuation-driven regime output rates are quite sensitive to input noise correlations (parameterized by the Fano factor), while in the mean-driven regime sensitivity is less pronounced.

In simulations of ring networks in the strongly fluctuation-driven case we observe that rates decrease with increasing correlations, while in otherwise equivalent random networks, rates increase (not shown). So the different recurrent input structure plays a crucial role for the effective input current and thus network dynamics properties. These differences are often, as here for ring and grid networks, a direct consequence of complex network topology, and it is thus important to understand how connectivity structure translates to single neuron activity, as well as collective network states. A more thorough analysis of such effects in the context of firing rate stability in random and spatially structured networks will be subject of subsequent research.

### 4.2. Impact of finite time scales

We assumed throughout the manuscript that the system has a negligible refractory time constant, small delays and instantaneous post-synaptic currents (δ-synapses). If these assumptions are dropped we observe differences especially for the mean-driven case: for larger delays *d* in the order of several milli-seconds, pronounced oscillations can occur that tend to synchronize the system locally on the order of the footprint (Kriener et al., 2009), or for very strong external drive also on a population-wide level (Brunel, 2000). A systematic study of the complex effects of delays on pattern formation in neural-field-type ring networks of spiking neurons was presented in Roxin et al. (2005). In the network we analyzed here, noisy delay oscillations mainly perturb developing patterns and can thus shift the onset of clear pattern formation to larger *J*_*c*_.

Similar effects arise in the case of finite refractory time τ_ref_. A larger τ_ref_, especially in the presence of oscillations, i.e., highly coincidental input spiking, decreases the effective network input, in particular if τ_ref_ > *d*, and thus also shifts the input regime. This can be compensated for by explicitly taking into account τ_ref_ in the derivation of the noiseless linearization.

Finally, if synaptic time constants are finite, while total input currents stay the same, delay oscillations can be smoothened out, and the effect of absolute refractoriness is decreased. Increasing synaptic time constants can thus counteract the effect of finite τ_ref_ and *d*.

### 4.3. Spiking networks vs. neural field models

Pattern formation, such as activity bump formation or periodic patterns, is a well-studied phenomenon in ring and toroidal networks (see e.g., Ermentrout and Cowan, 1979a,b; Ben-Yishai et al., 1995; Usher et al., 1995; Bressloff and Coombes, 1998, 2000; Shriki et al., 2003; Marti and Rinzel, 2013). Earlier studies of periodic pattern formation in spiking neuron networks already showed that ring- and grid-networks of mean-driven neurons can undergo a Turing-bifurcation (Usher et al., 1995; Bressloff and Coombes, 1998, 2000). In particular, Usher et al. (1995) observed a dependence on the input level. For weaker, yet suprathreshold mean input current, activity patches diffuse chaotically across the spatial extend of the system, while for stronger inputs a stable spatial pattern develops that relates to the critical eigenmode. We observe similar dynamics in the networks considered here as well, if the system is in the intermediate regime. This can be understood as follows: if the noise component is relatively strong, it shifts the actual critical coupling strength *J*^md^_*c*_ to higher values *J*^inter^_*c*_ inbetween *J*^md^_*c*_ and *J*^fd^_*c*_. If the system is thus effectively slightly sub-critical in terms of *J*^inter^_*c*_, input-noise can transiently excite eigenmodes close to effective criticality, due to Hebbian (Dayan and Abbott, 2001) or non-normal amplification (Murphy and Miller, 2009).

To compare our results to the classical work on neural field models on ring topologies (Ermentrout and Cowan, 1979a,b, 1980) we moreover studied a coarse-grained model that reduces the *N*-dimensional system to an effectively two-dimensional one that is structurally equivalent to the linearized two-population model presented, e.g., in (Ermentrout and Cowan, 1980) (see supplementary material section S2) which, however, allows for a direct quantitative mapping to the spiking network dynamics.

Because of the coarse-graining some of the connectivity details get lost and thus the critical eigenmode is not identical with that of the original ring network. Moreover, we observe the importance of excluding self-coupling in the coarse-grained model: if that is not excluded the resulting eigensystem looks dramatically different with nearly completely real-valued eigenvalues.

### 4.4. Impact of structural noise

For the translation-invariant distribution of inhibitory neurons across a ring topology the eigensystem, and hence the respective linear stability, can be computed analytically. If inhibitory neurons are however randomly distributed, some parts of the ring will have a higher, others a lower density of inhibitory neurons, such that these networks form an ensemble of possible realizations, most of which are not invariant to translations. The resulting inhomogeneous rate per neuron in the subcritical regime can still be predicted well from the linear rate model. However, even when knowing the dominant eigenvector, it will usually not be periodic, and due to the inhomogeneous input per neuron different neurons will be at quite different working points. There is interference between the rate distribution and the dominant mode. Thus, the activity pattern that evolves in the supracritical regime is not straightforward to predict.

If the weights are distributed fully randomly across the whole ring topology irrespective of the identities of the neurons, i.e., in violation of Dale's principle, the resulting eigensystems look very similar to those of corresponding networks with random topologies. Only a few surviving singular eigenvalues serve to distinguish the underlying ring from the random topology. This directly translates to the spiking dynamics: the activity is much more asynchronous than that of Dale-conform networks and most of all pattern formation can not take place due to the effective lack of lateral inhibition.

### 4.5. Impact of network size

If κ/*N* is kept constant when varying the network size *N* the critical coupling strength in both the mean- and the fluctuation-driven regime decreases. For example, for *N* = 10000 and κ = 1000 the critical coupling strength becomes *J*^md^_*c*_ ≈ 0.2 mV for the mean-driven, and *J*^fd^_*c*_ ≈ 0.32 mV for the fluctuation-driven regime. If the number of synapses per neuron κ is fixed when *N* increases, both *J*^md^_*c*_ and *J*^fd^_*c*_ hardly change because the maximal eigenvalue is approximately unaffected by network dilution.

We emphasize that the analysis presented here can be extended to two-dimensional grid networks in a straightforward manner, see supplementary material section S3. We conclude by remarking that the rate model Equation (13) is also a valuable tool to study the rate dynamics of spatially embedded spiking neuron networks with e.g., inhomogeneous neuron distribution and distance-dependent connectivity in the mean-driven regime, allowing for a treatment of more general networks than commonly studied random networks or neural field models. The presented model is in conclusion a further step in the efforts to find and understand appropriate descriptions of the high-dimensional spiking activity in structured networks.

### Conflict of interest statement

The authors declare that the research was conducted in the absence of any commercial or financial relationships that could be construed as a potential conflict of interest.

## Footnotes

^1^Note, that for exponential response kernels *h*(*t*) = τ exp[−*t*/τ] the response is structurally equivalent to a linear first order differential equation, though it has been demonstrated that in ring networks this response kernel can be substantially different from exponential and even contain oscillatory components (Lindner and Schimansky-Geier, 2001; Pernice et al., 2012).

## A. Appendix

### A1. Model and simulation description

Table A1 **Model description, simulation paradigm and parameters**.

**Table:**

**A**	**MODEL SUMMARY**
Populations	Three: excitatory, inhibitory, external input
Connectivity	Ring connectivity with excitatory and inhibitory neurons arranged on the same ring, such that each fifth neuron is inhibitory; same connection footprint for both populations
Neuron model	Leaky integrate-and-fire (LIF), fixed voltage threshold precise spike timing (Hanuschkin et al., 2010)
Synapse model	δ-current inputs (discontinuous voltage jumps)
Input	Independent Poisson spike trains

**Table:**

**B**	**POPULATIONS**	
**Name**	**Elements**	**Size**
E	LIF neuron	*N*_E_ = 4*N*_I_
I	LIF neuron	*N*_I_
E_x_	excitatory Poisson input generator	one realization per neuron
I_x_	inhibitory Poisson input generator (only for the fluctuation-driven case)	one realization per neuron

**Table:**

**C**	**CONNECTIVITY**		
**Name**	**Source**	**Target**	**Pattern**
EE	E	E	connect to all excitatory neurons within a given distance on the ring, weight *J*, delay *d*
IE	E	I	connect to all excitatory neurons within a given distance on the ring, weight *J*, delay *d*
EI	I	E	connect to all excitatory neurons within a given distance on the ring, weight −*gJ*, delay *d*
II	I	I	connect to all excitatory neurons within a given distance on the ring, weight −*gJ*, delay *d*
E_x_	E_x_	E ∪ I	independent Poisson spike trains, weight *J*_x_, rate ν_Ex_
I_x_	I_x_	E ∪ I	independent Poisson spike trains, weight −*gJ*_x_, rate ν_Ix_

**Table:**

**D**	**NEURON AND SYNAPSE MODEL**
Name	LIF neuron
Type	Leaky integrate-and-fire, δ-current input
Subthreshold dynamics	$\begin{array}{l} \tau_{\text{m}}\dot{V}(t)=-V(t)+RI(t)\text{ if }t>t^{*}+\tau_{\text{ref}}\\ \;V(t)=V_{\text{res}}\text{ else}\\ \;RI(t)=\sum_{j=1,i\neq j}^{N+N_{\text{ext}}}\tau_{\text{m}}W_{ij}\sum_{k}\delta (t-t_{i,k}-d)+RI_{\text{x}}(t),\;W_{ij}\;\in \{-gJ,0,J\} \end{array}$
Spiking	If *V*(*t*−) < θ ∧ *V*(*t*+) ≥ θ
	1. set *t*^*^ = *t*
	2. emit spike with time-stamp *t*^*^

**Table:**

**E**	**INPUT**
**Type**	**Description**
Poisson generators	Rate ν_Ex_, ν_Ix_, each generator projects independent realizations to all neurons

### A2. Approximation of the siegert formula by an affine-linear input-output relation

For strong mean drive μ[*RI*] » θ we can approximate the input-output relation (10) by an affine linear relationship. In the following we assume the firing rate 0 < ν « 1/τ_ref_, which is the case for all relevant regimes, so we can neglect the effect of the refractory time τ_ref_. We use the Ansatz

(A1) $$\begin{array}{l} \nu (I)=-{\left(\tau_{\text{m}}\text{Log}\left[1-\theta /(RI)\right]\right)}^{-1}≐a+bRI\\ \;\Leftrightarrow -\tau_{\text{m}}\text{Log}\left[1-\theta /(RI)\right]≐{\left(a+bRI\right)}^{-1} \end{array}$$

By taking the derivative with respect to *RI* on both sides of the last expression we obtain

(A2) $$\begin{array}{l} -\frac{\tau_{\text{m}}}{1-\theta /(RI)}\theta /{\left(RI\right)}^{2}=-b{\left(a+bRI\right)}^{-2}\\ \;\frac{\theta \tau_{\text{m}}}{\theta RI-{\left(RI\right)}^{2}}=\frac{-1}{a^{2}/b+2aRI+b{\left(RI\right)}^{2}} \end{array}$$

Equating the terms in proportion to (*RI*) and in proportion to (*RI*)^2^ in the denominator fixes the parameters $a=-\frac{1}{2\tau_{\text{m}}}$ and $b=\frac{1}{\theta \tau_{\text{m}}}$, leading to the approximation

(A3) $$\nu (RI)≐-\frac{1}{2\tau_{\text{m}}}+\frac{1}{\theta \tau_{\text{m}}}RI$$

Indeed, to show that for θ » *RI*

(A4) $$-\frac{1}{\tau_{\text{m}}\text{Log}\left[1-\frac{\theta }{RI}\right]}≐-\frac{1}{2\tau_{\text{m}}}+\frac{1}{\theta \tau_{\text{m}}}RI.$$

we introduce the auxiliary variable θ/*RI* =: (1 − *x*) and compute the limit

(A5) $$\underset{x↗1}{\lim}\left[\frac{1}{\text{Log}[x]}+\frac{1}{1-x}\right]=\underset{x↗1}{\lim}\left[\frac{1-x+\text{Log}[x]}{\text{Log}[x](1-x)}\right].$$

Both numerator and denominator of this expression tend to zero, thus we apply L'Hôpital's rule and obtain for (A5)

(A6) $$\underset{x↗1}{\lim}\left[\frac{1/x-1}{1/x-1-\text{Log}[x]}\right].$$

Again, numerator and denominator tend to zero, thus we apply L'Hôpital's rule again and obtain for (A6)

(A7) $$\underset{x↗1}{\lim}\left[\frac{1/x^{2}}{(1+x)/x^{2}}\right]=\underset{x↗1}{\lim}\left[\frac{1}{1+x}\right]=\frac{1}{2}.$$

This yields (A4).

As a heuristic observation, we note moreover, that in the large-σ_*o*_-limit, i.e., σ_*o*_ » μ_*o*_, the integrand of Equation (5) becomes basically unity, and the integral thus scales as $\nu_{o}^{-1}\sim \sqrt{\pi }\tau_{\text{m}}\theta /\sigma_{o}$. In particular we observe that the input-output-relation Equation (5) for *V*_res_ = 0 takes the following linear form in σ_*o*_:

(A8) $$\nu_{o}(\sigma_{o},\mu_{o})=\frac{1}{\tau_{\text{m}}\sqrt{\pi }}\left(\frac{\sigma_{o}+\mu_{o}}{\theta }-\frac{1}{\pi }\right).$$

The input-output-relation of the LIF is thus also linear in σ_*o*_ for σ_*o*_ » μ_*o*_, see supplementary material section S4.

### A3. Eigenspectrum of the ring graph with equidistant inhibitory neurons

We consider graphs, in which each neuron *i* is connected to neurons *i* − κ/2 to *i* + κ/2, κ even, but not to itself. To keep analysis simple, we assume that each ℓ-th neuron, *N* mod ℓ = 0 is inhibitory.

Define *T*_ℓ_ as the translation operator that shifts all neuron indices by ℓ such that for every vector *v* we have

(A9) $$\begin{array}{l} T_{\ell }v=T_{\ell }{\left(v_{0},v_{1},v_{2},...,v_{N-1}\right)}^{⊤}\\ \;={\left(v_{\ell },v_{\ell +1},v_{\ell +2},...,v_{N-1},v_{0},...,v_{\ell -1}\right)}^{⊤} \end{array}$$

i.e., for the node index *n*

(A10) $$T_{\ell }[n]=(n+\ell )\text{mod}N,n\in \{0,...,N-1\}.$$

*T*_ℓ_ is a unitary operator and has eigenvalues φ_*l*_ and eigenvectors *w*_*l*_, such that

(A11) $$T_{\ell }w_{l}=e^{i\alpha_{l}}w_{l}=:\varphi_{l}w_{l},$$

with

(A12) $$T_{\ell }^{N/\ell }w_{l}=w_{l}\Rightarrow \alpha_{l}=2\pi l\ell /N,l\in \{0,...,N/\ell -1\}.$$

Hence the *N*/ℓ ℓ-fold degenerated eigenvalues of *T*_ℓ_ are φ_*l*_ = *e*^*i*2π*l*ℓ/*N*^, *l* ∈ {0, …, *N*/ℓ − 1}. The eigenvectors *w*_*l*_ are discrete harmonics and fullfil

(A13) $$w_{l}{}_{{}_{(i+\ell )\text{mod}N}}=\varphi_{l}w_{l}{}_{{}_{i\text{mod}N}}.$$

We define

(A14) $${\mathbb{C}}^{\ell }∋\tilde{w}={\left(w_{0},w_{1},w_{2},w_{3},...,w_{\ell -1}\right)}^{T}$$

and the map η that embeds the ℂ^ℓ^ as a subspace into the ℂ^*N*^

(A15) $$\eta :{\mathbb{C}}^{\ell }\to {\mathbb{C}}^{\ell }\subset {\mathbb{C}}^{N},\tilde{w}\mapsto \sum_{i=0}^{\ell -1}{\tilde{w}}_{i}{\text{e}}_{i}=:\tilde{v},$$

where *e*_*i*_, *i* ∈ {0, …, *N* − 1} are the *N* canonical basis-vectors of the ℝ^*N*^ and $\tilde{w}$_*i*_ ∈ ℂ is the *i*-th vector component of $\tilde{w}$. We moreover define the operator family *P*_*l*_, *l* ∈ {0, …, *N*/ℓ − 1}

(A16) $$P_{l}:{\mathbb{C}}^{\ell }\subset {\mathbb{C}}^{N}\to {\mathbb{C}}^{N},\tilde{v}\mapsto \sum_{j=0}^{N/\ell -1}T_{\ell }^{j}e^{-i2\pi jl\ell /N}\tilde{v}.$$

We see that (*P*_*l*_ ◦ η) indeed projects into the eigenspaces and is an isomorphism

(A17) $$P_{l}◦\eta :{\mathbb{C}}^{\ell }\to \text{Eig}(T_{\ell },\varphi_{l})\subset {\mathbb{C}}^{N}$$

as can be readily checked:

We see that for each *l* ∈ {0, …, *N*/ℓ − 1}

(A18) $$\begin{array}{l} T_{\ell }(P_{l}◦\eta )=T_{\ell }\sum_{j=0}^{N/\ell -1}T_{\ell }^{j}e^{-i2\pi jl\ell /N}=\sum_{j=0}^{N/\ell -1}T_{\ell }^{j+1}e^{-i2\pi jl\ell /N}\\ \;\overset{\left(*\right)}{=}e^{i2\pi l\ell /N}\sum_{k=0}^{N/\ell -1}T_{\ell }^{k}e^{-i2\pi kl\ell /N}=\varphi_{l}(P_{l}◦\eta ), \end{array}$$

where in the equation marked by (^*^) we performed an index shift and used (*e*^−*i*α^*T*_ℓ_)^*N*/ℓ^ = (*e*^−*i*α^*T*_ℓ_)^0^.

Linearity:

(A19) $$\begin{array}{l} \forall_{v,w\in {\mathbb{C}}^{\ell },a\in \mathbb{C}}(P_{l}◦\eta )(aw)=a(P_{l}◦\eta )w\text{and}(P_{l}◦\eta )(w+v)\\ \;=(P_{l}◦\eta )w+(P_{l}◦\eta )v \end{array}$$

Injectivity:

$$\text{Ker}(P_{l}◦\eta )=\{0\}$$

Surjectivity:

$$\text{dim}[(P_{l}◦\eta )({\mathbb{C}}^{\ell })]=\text{dim}[\text{Eig}(T_{\ell },\varphi_{j})]=\ell .$$

Now, we can calculate the spectrum of *W*. Due to [*W, T*_ℓ_] = 0, i.e., *W* and *T*_ℓ_ commute, we can diagonalize both matrices, such that they have a common system of eigenvectors. Hence, it is sufficient to reduce the problem to the ℓ-dimensional vector-space embedded in ℂ^*N*^, spanned by eigenvectors of Eig(*T*_ℓ_, *e*^*i*φ_*l*_^)|_ℂ^ℓ^_, i.e., $\tilde{w}$_*l*_, *l* ∈ {0, …, *N*/ℓ − 1}. To obtain the eigenspaces Eig(*W*, λ_*l*_*i*__) we only need to solve the diagonalization problem for the corresponding (ℓ × ℓ)-matrices η^−1^ ◦ *W* ◦ *P*_*l*_ ◦ η = (*P*_*l*_ *W*)|_ℂ^ℓ × ℓ^_:

(A20) $$(\eta^{-1}◦W◦P_{l}◦\eta )\tilde{w}=\lambda \tilde{w}$$

Once we got the ℓ eigenvalues and eigenvectors {λ_*l, i*_, $\tilde{w}$_*l, i*_} of *W* in the respective sub-spaces, the operator *P*_*l*_ ◦ η generates the corresponding *N*-dimensional eigenvector *w*_*l*_.

### A4. Eigensystem of the reduced ring system

We now consider a coarse-grained network model where connectivity is defined between *C* = *N*/ℓ cells of ℓ neurons each as depicted in Figure 6A. Each neuron is connected to all other ℓ − 1 neurons within the cell it resides in, as well as to all neurons in the neighboring 2*K* = *pC* cells, yielding block-wise connectivity matrices as indicated in Figure 7B. We introduce a new global neuron labeling such that

(A21) $$n=c\ell +i_{c},\;c=\left\lfloor \frac{n}{\ell }\right\rfloor ,\text{ and }i_{c}=n\text{mod}\ell$$

**Symmetries**:

The system in invariant to shifts about elementary cells, i.e., for the cell index *n* we have again invariance to the operation

(A22) $$T_{\ell }[n]=(n+\ell )\text{mod}N.$$

Moreover, there are two mirror symmetries *R* and *S* within each cell (cf. Figures 6B,C), such that

(A23) $$Rw_{\left[n\right]}=Rw_{\left[(c\ell +i_{c})\text{mod}N\right]}=\{\begin{array}{ll} w_{\left[(c\ell +(1-i_{c}))\text{mod}N\right]} & \text{if }i_{c}\leq 1\\ w_{\left[n\right]} & \text{if }i_{c}>1 \end{array}$$

(A24) $$Sw_{\left[n\right]}=Sw_{\left[(c\ell +i_{c})\text{mod}N\right]}=w_{\left[\left(c\ell +\ell -1-i_{c}\right)\text{mod}N\right]}$$

*R* and *S* are operators, such that for the eigenvalues *r*_*j*_ and *s*_*j*_, and for the eigenvectors *v*_*j*_ and *w*_*j*_ we have *R*^2^*v*_*j*_ = *r*^2^_*j*_*v*_*j*_ = *v*_*j*_ and *S*^2^*w*_*j*_ = *s*^2^_*j*_*w*_*j*_ = *w*_*j*_. Hence, *r*_*j*_, *s*_*j*_ ∈ {−1, 1}. We have three commuting operators *T*_ℓ_, *R, S*, i.e.,

$$[T_{\ell },R]=[T_{\ell },S]=[S,R]=0.$$

So, we can find a common basis of eigenvectors to span the complete system. For each wavenumber Λ_*i*_ = α_*i*_ *N*/2πℓ one eigenvector is obviously given by

(A25) $$v_{\alpha_{i}}^{\text{I}}:=\sum_{c=0}^{N/\ell -1}{\left(e^{-i\alpha_{i}}T_{\ell }\right)}^{c}v_{(\ell -1)/2}e_{(\ell -1)/2}$$

where *e*_(ℓ − 1)/2_ is the (ℓ − 1)/2-th canonical basisvector of the ℝ^*N*^ and (ℓ − 1)/2 is the index of the first inhibitory neuron on the ring enumerated as in Figure 6, since this dimension does not couple to either *R* or *S*. To get the residual *N* − *N*_*I*_ eigenvectors, we choose

(A26) $$v_{r,s,\alpha_{i}}^{\text{E}}:=\sum_{c=0}^{C-1}{\left(e^{-i\alpha_{i}}T_{\ell }\right)}^{c}(1+sS)(1+rR)v_{0}e_{0}$$

with first canonical basis vector *e*_0_.

Indeed, we see again that for each *c* ∈ {0, …, *N*/ℓ − 1}

(A27) $$\begin{array}{l} T_{\ell }{\left(e^{-i\alpha }T_{\ell }\right)}^{c}=e^{-i\alpha c}T_{\ell }^{c+1}=e^{i\alpha }e^{-i\alpha (c+1)}T_{\ell }^{c+1}\\ \;=e^{i\alpha }{\left(e^{-i\alpha }T_{\ell }\right)}^{c+1}\\ \text{ and }{\left(e^{-i\alpha }T_{\ell }\right)}^{C}=1, \end{array}$$

and  with *x* ∈ {*r, s*} and  ∈ {*R, S*}, so *v*_*r, s*, α_ are eigenvectors with eigenvalues *r, s*, α. Evaluation of the eigenvectors for the two possible eigenvalues of *R, S* each yields

(A29) $$\begin{array}{l} \;v_{1,1,\alpha }^{\text{E}}=\sum_{c=0}^{C-1}{\left(e^{-i\alpha }T_{\ell }\right)}^{c}({\left(v_{0},v_{0},0,v_{0},v_{0},0,\ldots ,0\right)}^{⊤})\\ \;v_{-1,1,\alpha }^{\text{E}}=\sum_{c=0}^{C-1}{\left(e^{-i\alpha }T_{\ell }\right)}^{c}({\left(v_{0},-v_{0},0,-v_{0},v_{0},0,\ldots ,0\right)}^{⊤})\\ \;v_{1,-1,\alpha }^{\text{E}}=\sum_{c=0}^{C-1}{\left(e^{-i\alpha }T_{\ell }\right)}^{c}({\left(v_{0},v_{0},0,-v_{0},-v_{0},0,\ldots ,0\right)}^{⊤})\\ v_{-1,-1,\alpha }^{\text{E}}=\sum_{c=0}^{C-1}{\left(e^{-i\alpha }T_{\ell }\right)}^{c}({\left(v_{0},-v_{0},0,v_{0},-v_{0},0,\ldots ,0\right)}^{⊤})\; \end{array}$$

### A5. Mean-field approximation of small-world networks of excitatory and inhibitory neurons

Analogous to the mean-field approach developed in (Grabow et al., 2012) for unweighted networks we will now compute the eigensystem of the mean-field small world network derived from the ring network with equidistant inhibition by random rewiring of edges (Kriener et al., 2009). In this case instead of assigning explicit weights *W*_*ij*_ ∈ {0, *J*, −*gJ*} to individual entries of the coupling matrix, all possible connections carry their expected weight as a function of the rewiring probability *p*_*r*_ such that all weights within the original ring neighborhood are scaled by *p*_1_(*p*_*r*_), and all weights that were 0 in the original ring are set to *W*_*ij*_ = *p*_2_(*p*_*r*_) *J* if *j* is excitatory, and *W*_*ij*_ = − *p*_2_(*p*_*r*_) *gJ* if *j* is inhibitory, where (cf. Kriener et al., 2009; Grabow et al., 2012)

(A30) $$\begin{array}{c} p_{1}(p_{r})=(1-p_{r})+\frac{p_{r}^{2}\kappa }{\left(N-1-\kappa (1-p_{r})\right)} \end{array}$$

$$p_{2}(p_{r})=\frac{p_{r}}{\left(N-1-\kappa (1-p_{r})\right)}$$

The resulting average coupling matrix is again invariant with respect to *T*_ℓ_ and the eigensystem can be determined exactly as described in Appendix A3.
