# Supplementary material for: How pattern formation in ring networks of excitatory and inhibitory spiking neurons depends on the input current regime
Source: Front Comput Neurosci. 2014 Jan 7;7:187. doi: 10.3389/fncom.2013.00187 (PMC3882721; doi:10.3389/fncom.2013.00187)
Supplement: Supplementary file 1 [file DataSheet1.PDF]

Supplementary material for

"How pattern formation in ring networks of excitatory and inhibitory spiking neurons depends on the input current regime"

Birgit Kriener, Moritz Helias, Stefan Rotter, Markus Diesmann, Gaute Einevoll

1 Relation of the coarse-grained model to Ermentrout-Cowan networks

In neural field modeling ring models are amongst the best-studied systems [1, 2, 3]. Neural field models are in principle derived from spiking neuron network dynamics by performing a continuum limit that yields neuron densities and spatial coupling kernels. In a common framework [5, 6, 1] the firing rate of a neuron k is given by a current $I_k(t)$ that non-linearly depends on the membrane potential by a transfer function $S(V_k(t))$, which is commonly assumed to be, e.g., a step function, a rectified linear function or a sigmoidal with activation threshold u. The input currents that arrive at a postsynaptic neuron i are then weighted, summed up and filtered by a temporal kernel $h(t)$ to give the membrane potential of the neuron, such that

$$V_i(t) = \int_{-\infty}^{t} h(t-\tau) \sum_k \alpha_{ik} S(V_k(\tau))\, d\tau + \int_{-\infty}^{t} h(t-\tau) P_i(\tau)\, d\tau\,, \tag{1}$$

where α_{ik} is the distance-dependent synaptic weight and $P(t)$ is an external current. If $h(t) = \exp[-t/\xi]/\xi$, the dynamics (1) can be transferred to a differential equation that is structurally equivalent to Eqn. (1) in the main manuscript, i.e.,

$$\xi \frac{dV_i(t)}{dt} = -V_i(t) + \sum_k \alpha_{ik} S(V_k(t)) + P_i(t)\,. \tag{2}$$

With $\alpha_{ik} = \alpha w(|i-k|)$, $\sum_k w(|k|) = 1$ the continuum limit then leads to the coupled field equations

$$\xi_{\mathcal{X}} \frac{dV_{\mathcal{X}}(x,t)}{dt} = -V_{\mathcal{X}}(x,t) + \sum_{\mathcal{Y}\in\{\mathcal{E},\mathcal{I}\}} \alpha_{\mathcal{XY}} \int_{-\infty}^{\infty} W_{\mathcal{XY}}(|x-x'|) S(V(x',t))\, dx' + P_{\mathcal{X}}(x,t)\,,\ \ \mathcal{X},\mathcal{Y} \in \{\mathcal{E},\mathcal{I}\}\,. \tag{3}$$

for two populations $\mathcal{E}$ (excitatory) and $\mathcal{I}$ (inhibitory). Stability analysis [1] leads to a linearized dynamical system and the eigenvalues $\lambda_H(\Lambda)$ of the matrix

$$H(\Lambda) = \begin{pmatrix} S'_{\mathcal{E}}(u)\alpha_{\mathcal{EE}}\hat{w}(\Lambda) - 1 & S'_{\mathcal{E}}(u)\alpha_{\mathcal{EI}}\hat{w}(\Lambda) \\ S'_{\mathcal{I}}(u)\alpha_{\mathcal{IE}}\hat{w}(\Lambda) & S'_{\mathcal{I}}(u)\alpha_{\mathcal{II}}\hat{w}(\Lambda) - 1 \end{pmatrix}, \tag{4}$$

– where $\hat{w}(\Lambda)$ denotes the Fourier transform of the coupling kernel – determines the stability of the spatially homogeneous mode (trivial solution) to spatially inhomogeneous perturbations of activity. In particular, for a boxcar coupling kernel $B(x,\sigma) := \Theta(|x|-\sigma)/2\sigma$ the Fourier transform is given by $\mathrm{sinc}(\sigma\Lambda) = \sin(\sigma\Lambda)/(\sigma\Lambda)$. If we assume that the transfer function is the same for all neurons, that $\alpha_{\mathcal{EE}} = \alpha_{\mathcal{IE}} = 4\alpha$, and $\alpha_{\mathcal{EI}} = \alpha_{\mathcal{II}} = -g\alpha$, and moreover substitute $S'(u) =: s'$, we obtain

$$H(\Lambda) = \begin{pmatrix} 4s'\alpha\,\mathrm{sinc}(\sigma\Lambda) - 1 & -gs'\alpha\,\mathrm{sinc}(\sigma\Lambda) \\ 4s'\alpha\,\mathrm{sinc}(\sigma\Lambda) & -gs'\alpha\,\mathrm{sinc}(\sigma\Lambda) - 1 \end{pmatrix}. \tag{5}$$

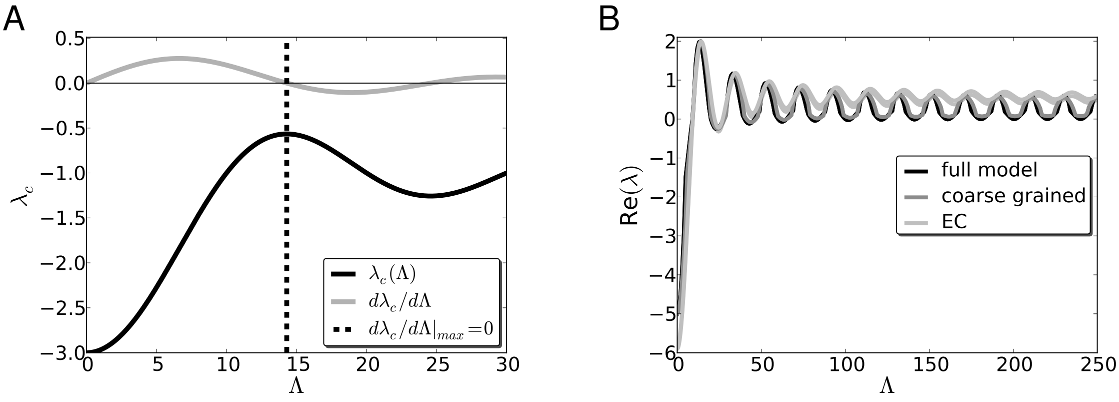

Figure 1: (A) The dispersion relation $\lambda_c(\Lambda)$ for the Ermentrout-Cowan (EC) model (black) and its derivative (gray) whose first zero gives the wavenumber of the global maximum. (B) The full model (black), the coarse-grained model (dark gray) and the EC model (light gray, here, S' was scaled to match the other two in maximal amplitude) in comparison.

With spatial extent $\sigma = 2\pi\kappa/N$ of the coupling kernel on two rings of circumference 2π and neuron densities $N_{\mathrm{I}}/2\pi$ and $N_{\mathrm{E}}/2\pi = 2N_{\mathrm{I}}/\pi$, respectively, we can thus map the ring model discussed previously to the neural field model of [1]. The resulting eigenvalues are $\lambda_H(\Lambda) = \{-1, -1 - 2\mathrm{sinc}(\sigma\Lambda)\}$, where $\mathrm{sinc}(x) = \sin(\pi x)/\pi x$. The latter eigenvalue $\lambda_c(\Lambda)$ gives the dispersion relation $f(\Lambda)$ whose maximum determines which wavenumber will grow after a spatial perturbation and is scaled and plotted in comparison to the respective relations from Secs. 3.3 and 3.4 in the main manuscript in Fig. 1 B. The maximum can be determined by finding the zero of the derivative of λ_c with respect to Λ, and is close to but not exactly an integer number (here ≈ 14.3, cf. Fig. 1 A), such that a rounding operation is needed to obtain the Fourier mode $v_c \sim e^{i\Lambda x}$ that complies with the constraint of periodic boundary conditions. We note that to relate the coarse-grained model to the classical result of Ermentrout and Cowan (1980), we specify the non-linearity in the latter work only up to the point that the derivative S' matches the linearized gain of the coarse-grained model in Fig. 1 A.

2 Comparison to two-dimensional torus-grids

Analogous to the way of computing the eigensystem in the one-dimensional case the eigensystem of a two-dimensional torus-grid can be computed in the following way.

The nodes of the network are distributed on a regular 2-dimensional grid with edge length N, such that $N \bmod 2 = 0$. We assume that each fourth neuron is inhibitory as indicated in Fig. 2 (A,) the gray dots mark the inhibitory, black ones the excitatory neurons, the boundary conditions are assumed to be periodic. The κ nearest neurons of a neuron i shall be neighbors of i with respect to the l^∞-norm, such that $\kappa = 4K(K+1)$, where K is the number of the quadratic shells surrounding each node. If we want $\kappa/N^2 \approx \epsilon \in [0,1]$, $K = \lfloor \frac{1}{2}\sqrt{1+\epsilon N^2} - \frac{1}{2} \rfloor$.

We will now solve the eigenvalue problem for the general case of a unit cell of size $M \times M$ with $M \leq N$ and $N \bmod M = 0$. The grid indices $\{i,j\},\ i,j = \{0,...,N-1\}$ map to the node indices n by

$$n = Ni + j,\ n \in \{0,...,N^2-1\},\ i = \left\lfloor \frac{n}{N} \right\rfloor,\ j = n \bmod N\,. \tag{6}$$

In grid indices we see that there are two invariant translation operations

$$T_i : \{i,j\} \to \{(i+M) \bmod N, j\} \tag{7}$$

and

$$T_j : \{i, j\} \to \{i, (j + M) \mathrm{mod}\, N\} \,. \tag{8}$$

Hence,

$$T_i[n] = N \left[\left(\left\lfloor \frac{n}{N} \right\rfloor + M \right) \bmod N \right] + n \bmod N \tag{9}$$

$$T_j[n] = N \left\lfloor \frac{n}{N} \right\rfloor + (n + M) \bmod N \tag{10}$$

The arbitrary translation about p cells in i- and q cells in j-direction across the grid is given by

$$T_i^p \, T_j^q \, [n] = N \left[\left(\left\lfloor \frac{n}{N} \right\rfloor + p\, M \right) \bmod N \right] + (n + q\, M) \bmod N \,. \tag{11}$$

We see that due to the symmetry properties of the system the coupling matrix W and the shifting operator $T_{p\,q} = T_i^p T_j^q$ commute, i.e., $[T_{p\,q}, W] = 0$ and so we can diagonalize both operators in a common basis of eigenvectors. The translation operators are unitary operators and thus have eigenvalues φ_l, ϑ_k and eigenvectors w_l, v_k, such that

$$\begin{aligned} T_i\, w_l &= e^{i\alpha_l}\, w_l = \varphi_l\, w_l \,, \\ T_j\, v_k &= e^{i\beta_k}\, v_k = \vartheta_k\, v_k \,. \end{aligned} \tag{12}$$

Due to the periodic boundary conditions the eigenvalues must fulfill

$$\begin{aligned} T_i^{N/M}\, w_l &= w_l \;\Rightarrow\; \alpha_l = 2\pi l M/N, \;\; l \in \{0, ..., N/M - 1\} \,, \\ T_j^{N/M}\, v_k &= v_k \;\Rightarrow\; \beta_k = 2\pi k M/N, \;\; k \in \{0, ..., N/M - 1\} \,. \end{aligned} \tag{13}$$

In our notation Eqn. 6, the node indices of the unit cell indices $m \in \{0, ..., M^2 - 1\}$ of the nodes marked by the red rectangle in Fig. 2 (A) (we start counting in the lower left corner and go on from left to right and finish in the upper right corner) are mapped to the global node index as $n = \iota(m)$, with

$$\iota \,:\, \{0, ..., M^2 - 1\} \to \{0, ..., N^2 - 1\}, \quad m \mapsto m \bmod M + N \left\lfloor \frac{m}{M} \right\rfloor \,. \tag{14}$$

Again we can define a map η from the M^2-dimensional space of the unit cell elements to the $\mathbb{C}^{M^2} \subset \mathbb{C}^{N^2}$

$$\eta \,:\, \mathbb{C}^{M^2} \to \mathbb{C}^{M^2} \subset \mathbb{C}^{N^2}, \quad \tilde{w} \mapsto w = \sum_{i=0}^{M^2-1} \tilde{w}_i\, \mathrm{e}_{\,\iota(i)} \tag{15}$$

with $\mathrm{e}_{\,i}$ the i-th canonical basis vector of the $\mathbb{R}^{N^2}$, $\tilde{w}$ the vector living on the sub-cell $M \times M$, and the operator family

$$P_{k\,l} = \sum_{p,q=0}^{N/M-1} T_{p\,q}\, e^{-i2\pi \frac{M}{N}(kp+lq)} \tag{16}$$

with $k, l \in \{0, ..., N/M - 1\}$.
$(P_{k\,l} \circ \eta)$ is an isomorphism

$$(P_{k\,l} \circ \eta) : \mathbb{C}^{M^2} \subset \mathbb{C}^{N^2} \to \mathrm{Eig}(T_{p\,q}, \varphi_l, \vartheta_k) \,, \tag{17}$$

as can be readily checked again. The effective eigenvalue problem is hence given by

$$(\eta^{-1} \circ W \circ P_{k\,l} \circ \eta)\tilde{w} = \lambda_{k\,l}\tilde{w}\,. \tag{18}$$

The eigenvectors of the full matrix W are again produced by applying $P_{k\,l} \circ \eta$ to the corresponding four-dimensional eigenvector of the reduced system.

For the two-dimensional embedding the grid-layout as sketched in Fig. 2 (A) was chosen (note however, that other grid embeddings can be dealt with in the same way). With this layout there are only 75% excitatory cells in the system, hence $\beta = 0.75$. For all other parameters as before, the two-dimensional system is less stable with regard to the coupling strength J if compared to the one-dimensional system, with inhibition being more dominant. However, if scaled to the same mean input, the torus grid is in general more stable with regard to increase in J.

3 Input-output relation is linear for large σ_o

As a heuristic observation, we note in the main manuscript, that in the large-σ_o-limit , i.e., $\sigma_o \gg \mu_o$, the integrand of Eqn. 19 becomes basically unity, and the integral thus scales as $\nu_o^{-1} \sim \sqrt{\pi}\tau_{\rm m}\theta/\sigma_o$. In particular, the input-output-relation

$$\nu_o^{-1} = \tau_{\rm ref} + \tau_{\rm m}\sqrt{\pi} \int_{\frac{V_{\rm res}-\mu_o}{\sigma_o}}^{\frac{V_{\rm thr}-\mu_o}{\sigma_o}} \exp\left[x^2\right]\,(1+{\rm erf}[x])\,dx, \tag{19}$$

for $V_{\rm res} = 0$ takes the following linear form in σ_o:

$$\nu_o(\sigma_o, \mu_o) = \frac{1}{\tau_{\rm m}\sqrt{\pi}}\left(\frac{\sigma_o + \mu_o}{\theta} - \frac{1}{\pi}\right)\,. \tag{20}$$

The validity of this approximation is demonstrated in Fig. 3.

References

[1] Ermentrout, B. and Cowan, J. (1980) Large scale spatially organized activity in neural nets. *SIAM Journal for Applied Mathematics*, 38, 1–21.

[2] Ben-Yishai, R., Bar-Or, R.L., Sompolinsky, H. (1995) Theory of orientation tuning in visual cortex. *Proceedings of the National Academy of Sciences*, 92, 3844–3848.

[3] Roxin, A., Brunel, N., and Hansel, D. (2005) The role of delays in shaping spatio-temporal dynamics of neuronal activity in large networks. *Physical Review Letters*, 94, 238103.

[4] Coombes, S. (2005) Waves, bumps, and patterns in neural field theories. *Biological Cybernetics*, 93, 91–108.

[5] Ermentrout, G. B. and Cowan, J. D. (1979) A mathematical theory of visual hallucination patterns. *Biological Cybernetics*, 34, 137–150.

[6] Ermentrout, G. B. and Cowan, J. D. (1979) Temporal oscillations in neuronal nets. *Journal of Mathematical Biology*, 7, 265–280.

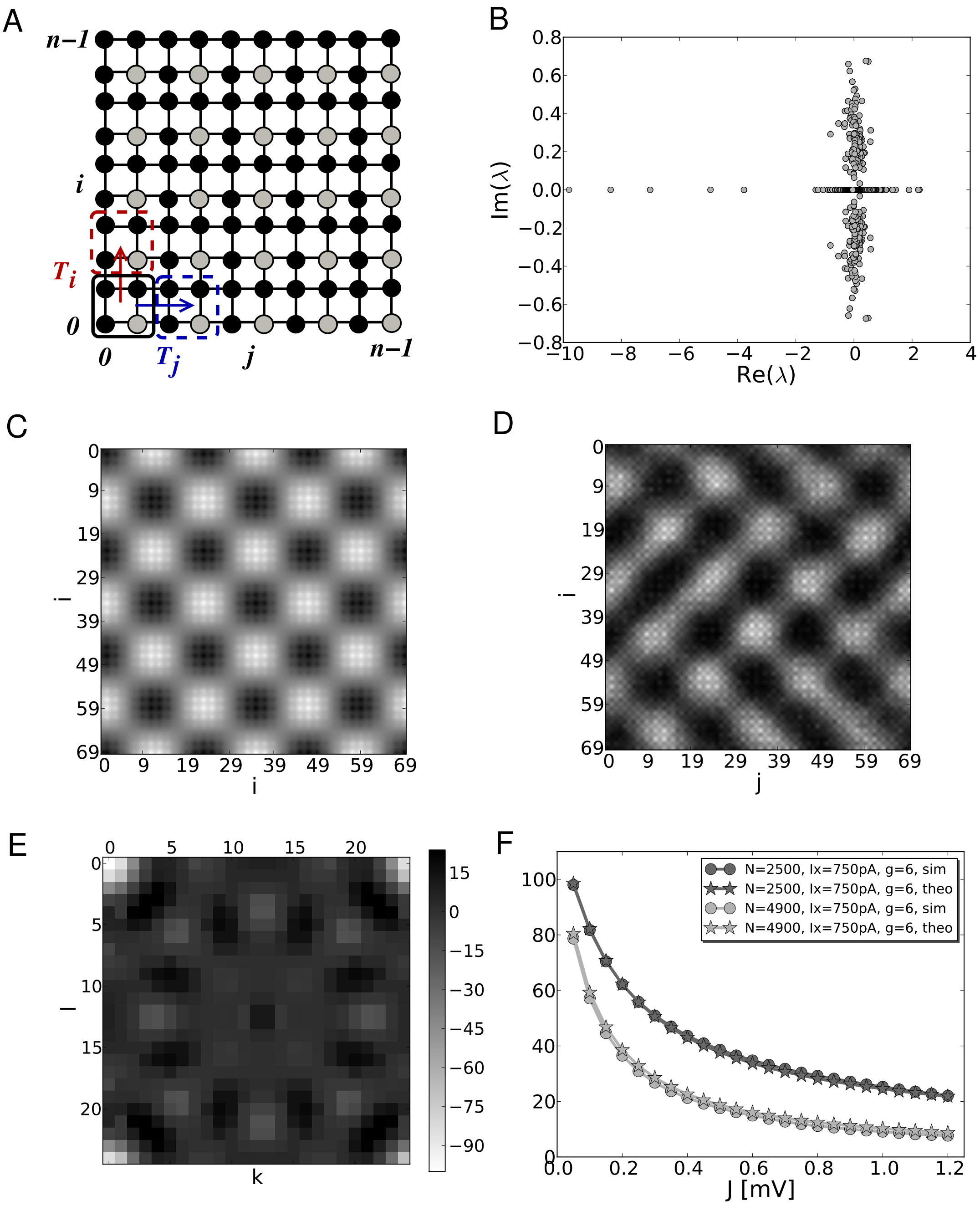

Figure 2: (A) Network layout of the torus grid network (gray dots: inhibitory, black: excitatory, red: minimal cell). (B) Eigenvalue spectrum of the rescaled coupling matrix for $N = 4900$ and $g = 6$. (C) Critical eigenvalue and (D) rate distribution in the supracritical regime. (E) shows the dispersion relation, i.e., the realpart of the eigenvalue as a function of the wavenumber. (F) shows the rate predictions from the simple linear rate model together with the simulation results.

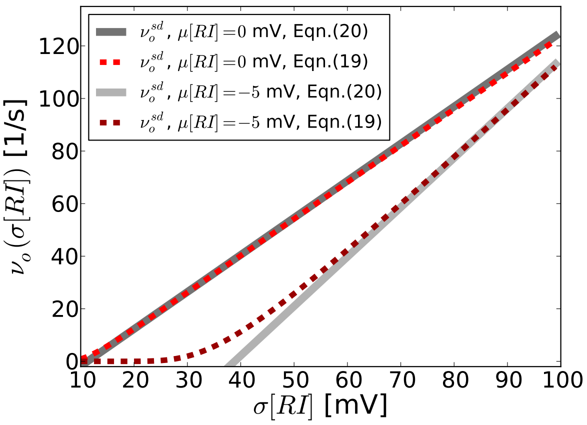

Figure 3: The figure demonstrates that the input-output-relation of the LIF neuron Eqn. 19 indeed gets linear also in the fluctuation-driven regime, cf. Eqn. 20. The gray lines show the linear predictions, while the red curves are the corresponding self-consistent rate given by Eqn. 19. Other parameters are $\theta = 20\,\mathrm{mV}$, $\tau_\mathrm{m} = 20\,\mathrm{ms}$, $\tau_\mathrm{ref} = 0.1\,\mathrm{ms}$ for $\mu = 0\,\mathrm{mV}$, while $\theta = 60\,\mathrm{mV}$, $\tau_\mathrm{m} = 5\,\mathrm{ms}$ for $\mu = -5\,\mathrm{mV}$.
